# Biomimetic 3D-Bioprinted organoids of thymic epithelial tumors for translational drug screening and biomarker identification

**DOI:** 10.1016/j.mtbio.2026.102878

**Published:** 2026-02-11

**Authors:** Beibei Liu, Huiyan Cheng, Keke Yu, Wen Xu, Xiaoting Tian, Yuhan Xu, Yanbin Kuang, Jun Lu, Rong Li, Xiao Zhang, Min Tang, Jianxin Xue, Yuqing Lou

**Affiliations:** a Department of Pulmonary Medicine, Shanghai Chest Hospital, Shanghai Jiao Tong University School of Medicine, Shanghai, 200030, China; b Shanghai Key Laboratory of Thoracic Tumor Biotherapy, Shanghai Chest Hospital, Shanghai Jiao Tong University School of Medicine, Shanghai, 200030, China; c Department of Pathology, Shanghai Chest Hospital, Shanghai Jiao Tong University School of Medicine, Shanghai, 200030, China; d Department of Laboratory Medicine, Zhongshan Hospital, Fudan University, Shanghai, 200032, China; e Department of Clinical Research Center, Tongren Hospital, Shanghai Jiao Tong University School of Medicine, Shanghai, 200336, China; f Institute of Interdisciplinary Integrative Medicine Research, Shanghai University of Traditional Chinese Medicine, Shanghai, 201203, China; g Department of Thoracic Oncology, Cancer Center, West China Hospital, Sichuan University, Chengdu, 610041, China

## Abstract

Thymic epithelial tumors (TETs), including thymic carcinoma and thymoma, are rare malignancies lacking both effective therapies and validated biomarkers to guide treatment. Here, we report the first 3D (three-dimensional) bioprinted organoid model of TETs, established through a proteomic data-driven biomaterial design strategy. Patient tumor tissues were first decellularized and analyzed by proteomics to determine their extracellular matrix (ECM) composition. The results revealed distributions of ECM proteins which guided the formulation of photocurable bioinks. The resulting 3D-bioprinted organoids supported primary TET cell proliferation, and more faithfully replicated the biophysical properties and molecular characteristics of native tumors than traditional Matrigel-cultured organoids. Leveraging this biomimetic platform, we conducted high-throughput drug screening and identified lurbinectedin as a potent therapeutic candidate for TETs. Transcriptomic profiling revealed its anti-TET mechanism. Integrating RNAseq data with TCGA survival analysis further identified PBX3, REPS2, and CXCR4 as potential efficacy-predictive biomarkers. This study establishes a translational framework linking 3D bioprinted TET models with biomarker discovery, offering a standardized platform for precision drug screening and mechanistic exploration in thymic epithelial tumors.

## Graphical abstract

**Figure:**
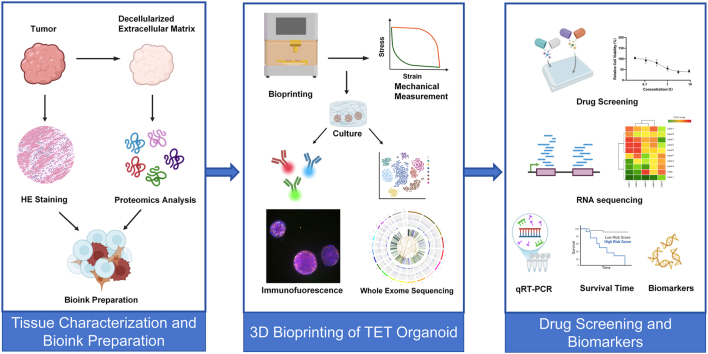

## 1 Introduction

Thymic epithelial tumors (TETs), including thymic carcinoma (TC) and thymoma (THYM), are rare but biologically heterogeneous malignancies arising from thymic epithelial cells [1]. There are significant differences in survival between different subtypes of TETs [2]. The 5-year overall survival rate of THYM is about 80%-90%, but the survival period of high-grade thymoma is significantly shortened. TC accounts for about 20-30%, including squamous cell carcinoma and neuroendocrine carcinoma [2,3]. Due to the relatively low incidence, molecular understanding of TETs is quite limited and has hindered progress in both targeted therapy development and biomarker discovery. Most patients rely on surgery and conventional platinum-based chemotherapy [4,5]. However, the overall objective response rate (ORR) to chemotherapy is below 40%, reflecting its limited efficacy [6]. Furthermore, no approved targeted drugs or validated biomarkers are currently available to guide precision management [5,7,8]. Driver mutations that confer sensitivity to existing targeted therapies, such as EGFR (Epidermal Growth Factor Receptor) or ALK (Anaplastic Lymphoma Kinase) alterations, are found in fewer than 5% of TET patients, leaving most TET patients without actionable molecular targets [9]. Therefore, there is an urgent need for mechanistically relevant in vitro models that can accelerate both therapeutic exploration and biomarker identification in TETs.

Organoids are three-dimensional (3D) cellular assemblies that better preserve the genetic heterogeneity, spatial organization, and microenvironmental interactions of primary tumors than conventional two-dimensional cell cultures [[10], [11], [12]]. However, traditional organoid systems remain limited by their dependence on Matrigel, an animal-derived basement membrane extract. Although Matrigel has been widely used in organoid research to mimic extracellular matrix (ECM) functions, its poor tunability, batch-to-batch variability, and low stiffness properties (elastic modulus ∼0.5-1 kPa) make it poorly representative of the stiffer tumor microenvironments typically found in solid malignancies [[13], [14], [15]]. These limitations substantially hinder reproducibility, quantitative control, and the accurate recapitulation of tissue-specific biophysical cues of organoids.

In recent years, 3D bioprinting has emerged as a next-generation organoid fabrication technology. Among the various 3D bioprinting modalities, digital light processing (DLP)-based bioprinting has attracted particular attention due to its cytocompatibility [16] and precise spatial and mechanical control of generated models [17,18]. Photocurable biomaterials combined with photoinitiator allows rapid fabrication of biomimetic tissue constructs using DLP bioprinting. GelMA (Gelatin Methacryloyl) preserves biocompatibility and integrin-binding motifs while enabling tunable stiffness through methacrylation. HAMA (Hyaluronic Acid Methacryloyl) provides hydrophilicity and viscoelasticity, mimicking the polysaccharide composition of natural ECM. In a previous study, 3D bioprinting technology was used to generate reproducible, biomimetic models of the complex brain tumor microenvironment [19]. In research on the relatively more common lung tumors, researchers constructed light-based 3D models by characterizing the physicochemical properties of the native extracellular matrix (ECM). These models can accurately recapitulate tissue interfaces and stiffness, thereby elucidating the effects of matrix stiffness on cell proliferation, invasion and drug sensitivity [20]. However, organoid studies on TETs remain extremely limited, with only a few reports using Matrigel-based cultures and no existing 3D-bioprinted organoid models reported to date [1]. The absence of such models underscores a critical unmet need for biomimetic, precisely engineered systems to investigate TET pathophysiology, drug response, and biomarker discovery in a reproducible manner.

In this study, we established the first proteomic profile of the ECM of TC and THYM tissues, providing valuable ECM landscape for future studies of TETs and offering guidance for material selection in in vitro model construction. Guided by this ECM information, we compared conventional Matrigel-based culture with a DLP printing strategy using composite bioink. The 3D-bioprinted TET organoids exhibited superior structural integrity, growth performance, and genomic fidelity, faithfully reproducing the histoarchitecture, driver mutations, and copy-number alteration patterns of primary tumors, thereby demonstrating high reproducibility and biological relevance. Building upon this biomimetic platform, we conducted high-throughput drug screening and identified lurbinectedin, a DNA topoisomerase II inhibitor approved for small-cell lung cancer, as a promising therapeutic candidate for TETs [[21], [22], [23]]. Subsequent comparative transcriptomic analyses between 2D and 3D models, integrated with molecular validation and TCGA survival correlation, revealed efficacy-predictive biomarkers that were associated with drug response. Together, these findings establish a biomarker-oriented 3D bioprinting framework that integrates ECM characterization, 3D model development, high-throughput screening, and biomarker identification and verification.

## 2 Results and discussion

### 2.1 Characterization of decellularized TETs reveals distinct ECM signatures

**Fig. 1:**
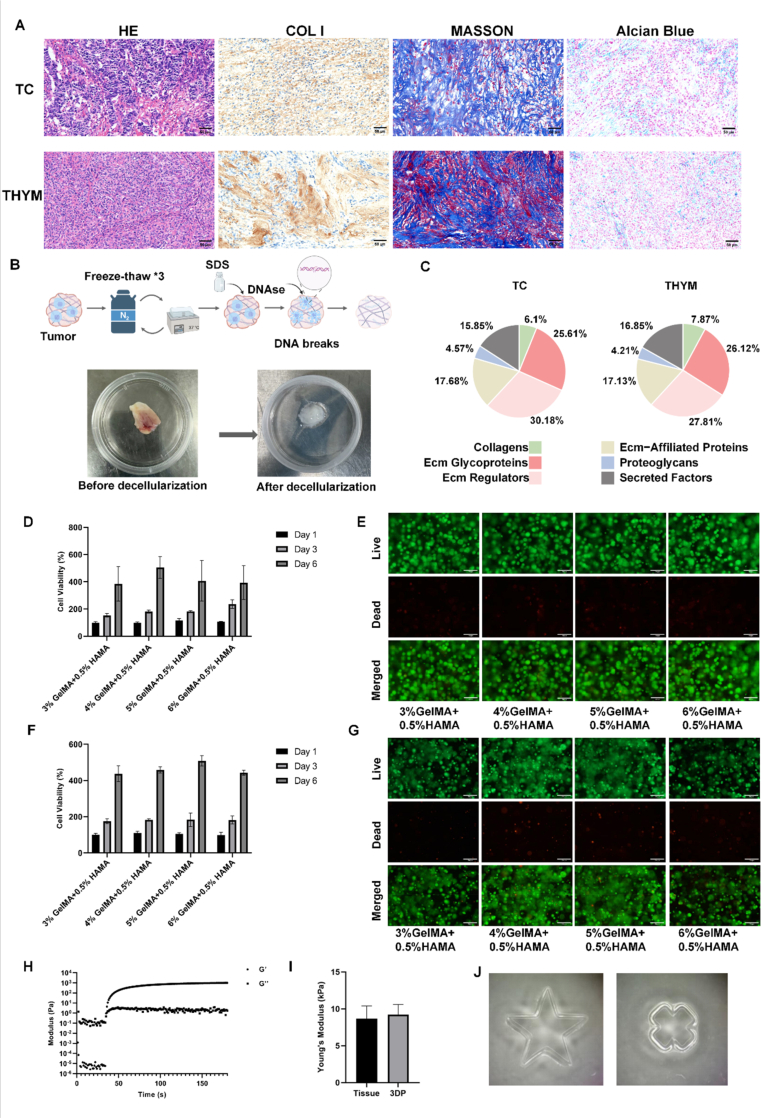
(A) Representative histological and immunohistochemical staining of TC and THYM tissues, including hematoxylin and eosin (HE), collagen type I (COL I), Masson's trichrome, and Alcian Blue staining.Scale bars: 50 μm (B) Schematic and gross images showing the decellularization process of TC and THYM tissues before and after treatment. (C) Proteomic classification of ECM components in decellularized TC and THYM matrices. (D-E) Cell viability of cells in 3D bioprinted constructs fabricated with different concentrations of GelMA (3%, 4%, 5%, 6%) supplemented with 0.5% HAMA was quantified via CTG assay at Day 1, Day 3, and Day 6, and visualized by live/dead fluorescence staining. (F-G) Cell viability of Ty-82 cells in 3D bioprinted constructs fabricated with different concentrations of GelMA (3%, 4%, 5%, 6%) supplemented with 0.5% HAMA was quantified via CTG assay at Day 1, Day 3, and Day 6, and visualized by live/dead fluorescence staining. (H) Rheological profiles showing the storage modulus (G′) and loss modulus (G″) of the 3DP bioink. (I) Comparison of Young's modulus among native tumor tissues, 3DP constructs, and Matrigel. (J) Optical images of 3DP GelMA–HAMA constructs with different geometries. (For interpretation of the references to color in this figure legend, the reader is referred to the Web version of this article.)

To enable biomimetic reconstruction of the tumor microenvironment, we first characterized the ECM composition of TC and THYM tissues. Histological staining (Fig. 1A and Supplementary Fig. 1A) revealed that both tumor types contained abundant type I collagen fibers, as confirmed by Masson's trichrome and COL I immunohistochemistry, whereas Alcian Blue staining indicated existence but relatively lower levels of hyaluronic acid (HA). These results suggest that the ECM of both TC and THYM is primarily collagen-rich with moderate HA content.

Following decellularization, tissues changed from firm, flesh-colored masses to soft, translucent matrices (Fig. 1B), confirming successful removal of cellular components while preserving ECM structure. As shown in Fig. 1C, similar proportions of structural proteins, such as collagens, glycoproteins, and proteoglycans, were identified in the ECM samples of TC and THYM. As shown in Supplementary Fig. 1B–C, functional enrichment analysis revealed that both the TC group and the THYM group exhibited enrichment of pathways linked to cell-matrix interaction and extracellular matrix (ECM) remodeling reflecting the close interaction between cells and ECM in the tumor microenvironment. The TC group showed significant enrichment in extracellular matrix remodeling, angiogenesis, and developmental programs, highlighting the tendency of dynamic matrix remodeling and angiogenesis, which indicates that the extracellular matrix of thymic carcinoma tumor cells has an invasive tendency [[24], [25], [26], [27]]. In contrast, the THYM group was enriched for inflammatory responses, secretory regulation, and immune modulation, consistent with thymic tissue's immunomodulatory and secretory functions [28], revealing tissue-specific functional specialization in microenvironment regulation. These molecular distinctions provide a mechanistic basis for the divergent biological behavior of the two TET types and guided the material selection strategy for subsequent organoid model construction.

Based on our histological and proteomic data, we fabricated bioinks using two materials, GelMA and HAMA. To better recapitulate the tumor extracellular matrix microenvironment, we conducted cell viability assays on 3D-printed models of the cell line. Firstly, bioink formulation tests were conducted in the IU-tab-1 cell line (derived from a type AB thymoma sample). Three groups were set up: single-component HAMA group, single-component GelMA group, and compound formulation group. For the single HAMA formulation (Supplementary Fig. 2A–B), the 0.5% HAMA group exhibited higher cell viability than the 1% HAMA group on day 6. It's indicate that 0.5% HAMA is more suitable for the culture of the tested cell line. This was consistent with histopathological staining results. For single-component GelMA formulations (Supplementary Fig. 2C–D), the 5% GelMA group was identified as the optimal single concentration. For the compound formulation (Fig. 1D and E), the highest cell viability was observed on day 6 when 0.5% HAMA was added to 4% GelMA, which was higher than that in the 5% GelMA single group and the 0.5% HAMA single group. However, the live/dead cell staining did not reveal any significant trend in the live/dead cell ratio. This indicates that the variation in CTG values between groups stems from differences in cell proliferation activity rather than cell apoptosis or necrosis induced by the bioink matrix.

Consistently, we repeated the same bioink formulation comparison experiments in the Ty-82 cell line (derived from an undifferentiated thymic carcinoma sample). Similar to IU-tab-1 cells, the 0.5% HAMA formulation demonstrates higher cell activity (Supplementary Fig. 2E–F). For the single-component GelMA group, results in Supplementary Fig. 2G–H indicated that 4% GelMA was the optimal concentration for Ty-82 cells. When 0.5% HAMA was combined with different concentrations of GelMA, CTG assay showed that the cell viability of the 5% GelMA +0.5% HAMA group was higher than that of other combinations (Fig. 1F and G). Notably, the CTG value of the 5% GelMA +0.5% HAMA group was lower than that of the single-component 4% GelMA group. Nevertheless, we ultimately selected 5% GelMA +0.5% HAMA for subsequent experiments, not solely based on cell proliferation reflected by CTG values. We comprehensively considered multiple factors, including the structural stability of the 3D model and the simulation effect of tissue stiffness. Additionally, based on the extracellular matrix (ECM) characteristics rich in glycoproteins, we also constructed traditional organoids based on Matrigel. Rheological and hardness tests demonstrated (Fig. 1H and I) that the hybrid material composed of 4-5% GelMA + 0.5% HAMA, when 3D photopolymerized, exhibited an elastic modulus highly consistent with that of natural tumor tissues, while Matrigel remained considerably softer [29,30]. Furthermore, this material could be fabricated into pre-designed stable structures (Fig. 1J) through this printing technology. These conditions significantly minimized experimental result deviations caused by structural damage to the models.

These data establish the first ECM proteomic landscape of TETs and demonstrate that 3D-bioprinted GelMA–HAMA constructs more closely replicate the mechanical stiffness of native tumors than Matrigel, providing a rational foundation for the subsequent construction and functional analysis of 3D TET organoid models.

### 2.2 Establishment and characterization of 3D-Bioprinted thymic tumor organoids

**Fig. 2:**
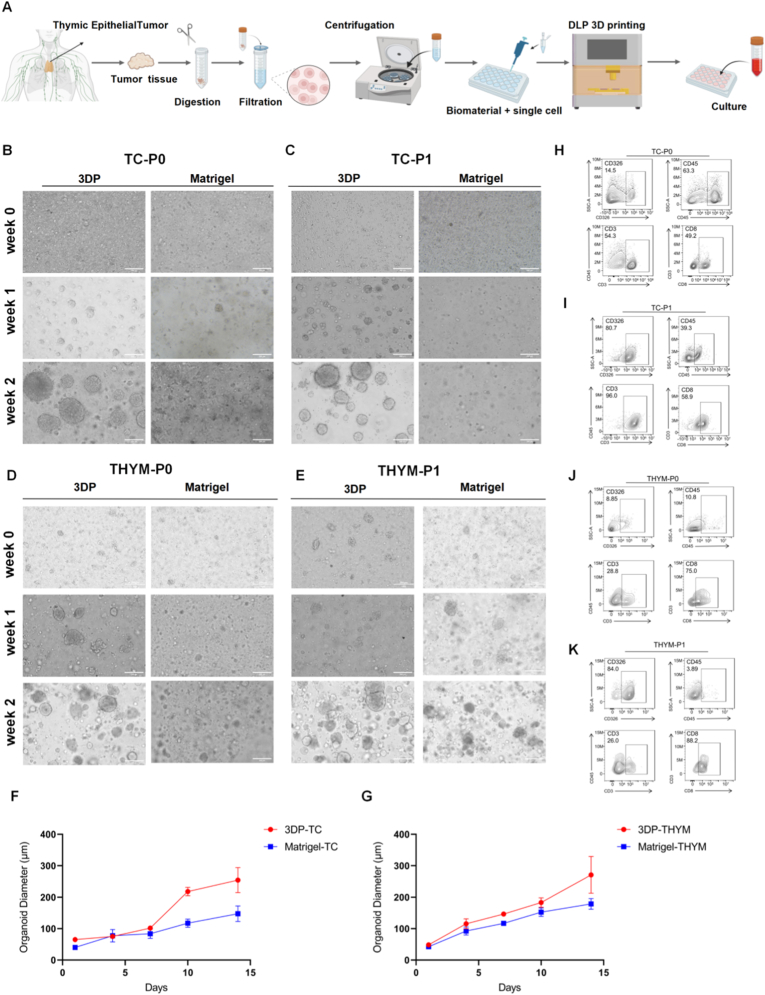
(A) Schematic illustration of the workflow for organoid generation, including tumor tissue dissociation, DLP–based 3D bioprinting, and in vitro culture. (B–E) Representative bright-field images showing the morphology of 3DP and Matrigel-cultured TC and THYM organoids at passages 0 (P0) and 1 (P1) from week 0 to week 2.Scale bars: 200 μm (F–G) Growth curves showing changes in average diameters of 3DP and Matrigel organoids derived from TC (F) and THYM (G) during 15 days of culture. (H–K) Flow-cytometric analysis of viable cells and marker expression (CD326, CD45) in TC (H–I) and THYM (J–K) organoids at P0 and P1.

To reconstruct thymic tumor models in vitro, tumor tissues from patients were dissociated into single-cell suspensions and incorporated into GelMA–HAMA bioink for DLP 3D bioprinting (Fig. 2A). The workflow consisted of three major stages: tissue processing, bioprinting construction, and subsequent culture. In parallel, Matrigel-based organoids were generated for comparison. Microscopic observations (Fig. 2B–E) showed that both 3D bioprinted (3DP) and Matrigel-embedded organoids from TC and THYM formed compact spherical or lobulated structures that increased in size over two weeks of culture. The 3DP organoids displayed more regular morphology and tighter cellular aggregation than those in Matrigel. 3DP models for TC and THYM could be serially passaged up to passage 5 while maintaining stable morphology (Supplementary Fig. 3A–B). Growth-curve analysis further confirmed sustained proliferative capacity, and the average diameters of 3DP organoids were slightly larger than those in Matrigel (Fig. 2F and G), indicating enhanced structural stability and long-term culture feasibility under the bioprinted condition.

To further assess cellular composition and viability, flow cytometry was performed to detect viable cells and the expression of key lineage markers CD326 (epithelial cells) and CD45 (immune cells) (Fig. 2H–K, Supplementary Fig. 3C). In TC organoids, from initial construction (P0) to post-passage (P1), the cell population displayed a consistent trend of increased viability, enrichment of epithelial cells, and reduction of immune cells. Specifically, viable cells increased from 57.2% at P0 to 84.8% at P1, reflecting improved culture stability. CD326^+^epithelial cells rose sharply from 14.5% to 80.7%, demonstrating successful enrichment of thymic epithelial-derived tumor cells. Meanwhile, CD45^+^ immune cells decreased from 63.3% to 39.3%, indicating that most non-tumor immune components were filtered out during culture while a portion of the immune microenvironment was retained. A similar pattern was observed in THYM organoids, which also exhibited progressive epithelial enrichment and immune cell reduction (Fig. 2J and K, Supplementary Fig. 3D). CD326^+^ epithelial cells increased markedly from 8.85% to 84.0%, consistent with the epithelial origin of THYM tumors, while CD45^+^ immune cells decreased from 10.8% to 3.89%.

Together, these results demonstrate that bioprinted TC and THYM organoids exhibit stable proliferation, preserved viability, and selective enrichment of tumor-derived epithelial cells, while retaining a fraction of immune components representative of the in vivo microenvironment. Compared with Matrigel-based culture, the 3D-printed system provides higher morphological fidelity and cellular consistency, establishing a robust and reproducible platform for downstream drug screening and biomarker discovery.

### 2.3 3D bioprinted organoids preserved tumor phenotypic and genomic fidelity

**Fig. 3:**
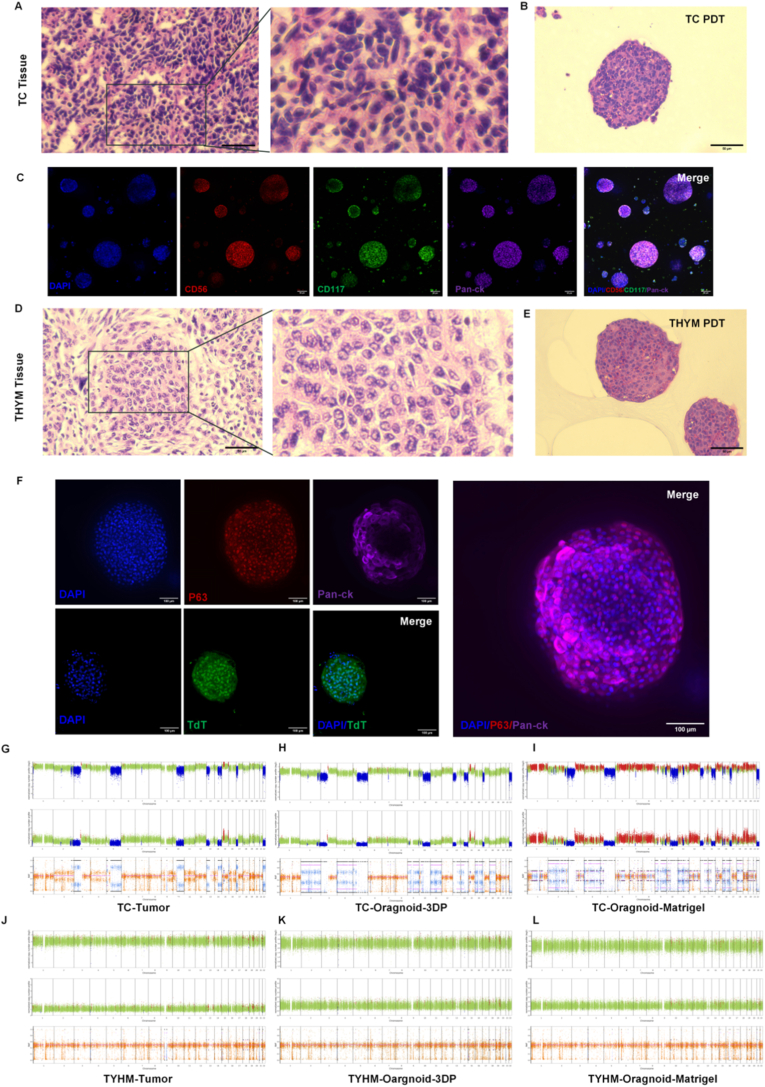
(A-B) H&E staining of primary TC tissue and corresponding 3DP organoids.Scale bars: 50 μm (C) Immunofluorescence images of TC organoids stained for DAPI, CD56, CD117, and pan-CK. Scale bars: 50 μm (D-E) H&E staining of primary THYM tissue and corresponding 3DP organoids. Scale bars: 50 μm (F) Immunofluorescence images of THYM organoids stained for DAPI, P63, pan-CK, and TdT.Scale bars: 100 μm. (G) CNV profiles of TC-Tumor. (H)TC-3DP organoids (I) TC-Matrigel organoids. (J) CNV profiles of THYM-Tumor. (K) THYM-3DP organoids. (L)THYM-Matrigel organoids.

To confirm that the 3D-printed organoids retained the phenotypic and genetic features of their parental tumors, we performed histological, immunofluorescent, and genomic analyses. H&E staining revealed that 3D-printed TC organoids recapitulated the dense cellular arrangement and high nuclear-to-cytoplasmic ratio characteristic of primary TC tissues (Fig. 3A and B). Immunofluorescence analysis showed strong CK expression, consistent with an epithelial origin, while CD56 and CD117 were also positive (Fig. 3C). CD117, a diagnostic marker distinguishing TC from THYM, confirmed the tumor subtype identity of the 3DP TC organoids. Similarly, the H&E pattern of 3D-printed THYM organoids matched that of the corresponding primary tissue, showing looser cellular organization (Fig. 3D and E). The marker panel for THYM organoids comprised DAPI, P63, CK, and TdT, selected according to the histopathologic profile of the parental tissue(Table 1). Immunofluorescence results demonstrated strong CK and P63 positivity, characteristic of epithelial thymoma, while TdT labeled reactive lymphocytes typically found in type A THYM (Fig. 3F). These findings confirm that both TC and THYM organoids maintained the histological and immunophenotypic traits of their respective tumors.

To evaluate genomic consistency, whole-exome sequencing (WES) was performed to detect copy-number variations (CNVs) and single-nucleotide variations (SNVs). The CNV profile of TC-Tumor (Fig. 3G) displayed characteristic deletions and amplifications in chromosomes 3p and 8q, regions frequently altered in TC [31,32]. The 3DP TC organoids (Fig. 3H) exhibited CNV patterns highly consistent with the parental tumor, preserving core amplification and deletion regions. In contrast, Matrigel-cultured TC organoids (Fig. 3I) retained the 3p deletion but displayed uneven 8q amplification and additional non-canonical gains, indicating lower replication fidelity. SNV analysis (Supplementary Fig. 4A) further confirmed that 3D-printed TC organoids reproduced the primary tumor's genomic landscape with higher accuracy. Core driver gene TP53 mutations were identical across TC-Tumor, TC-3DP, and TC-Matrigel. Pathway genes associated with neuroendocrine tumor proliferation and differentiation, such as NOTCH1 and FGFR3 [33], were concordant between TC-Tumor and TC-3DP, whereas TC-Matrigel organoids exhibited minor deviations in NOTCH1 mutation patterns. In THYM, the CNV profile of THYM-Tumor (Fig. 3J) revealed characteristic alterations at chromosomes 6q and 11q [31,34]. The 3D-printed THYM organoids (Fig. 3K faithfully reproduced these CNV signatures, while Matrigel-cultured organoids (Fig. 3L) showed an additional deletion signal at chromosome 5p absent in the parental tissue. SNV profiling (Supplementary Fig. 4B) demonstrated that mutations in key driver genes, such as SMARCD1, ARID1A, and JAK3 [31,35,36], were consistent between THYM-Tumor and THYM-3DP, whereas THYM-Matrigel organoids exhibited partial loss or altered mutation types, such as the disappearance of ARID1A or BCL11A variants. However, the SNVs of the two THYM organoid models were significantly different from the original tissues. This may be due to the significant intratumoral heterogeneity of thymoma tissue, which contains multiple clonal subpopulations with distinct mutation profiles. During organoid establishment, only some clonal subpopulations can adapt to the culture environment of in vitro Matrigel/bioink and expand successfully. This leads organoids to represent only partial clones of the original tissue, resulting in discrepancies between the single-nucleotide variants (SNVs) results and the primary tissue that includes the full clonal spectrum [37]. During organoid passaging, initial low-frequency mutations may be enriched into high-frequency mutations through random clonal expansion, or some mutations from the primary tissue may be lost due to clonal bottleneck effect, leading to dynamic changes in the SNV spectrum [38].

Collectively, these results demonstrate that 3D-printed TC and THYM organoids accurately preserve the histological phenotype, immunophenotypic markers, and genomic architecture of the original tumors, outperforming Matrigel-based cultures in maintaining genomic fidelity. This confirms the suitability of the 3D-printed model as a reliable platform for downstream drug testing and biomarker discovery [39,40].

### 2.4 Drug screening on bioprinted models revealed lurbinectedin as a potent compound for TETs

**Fig. 4:**
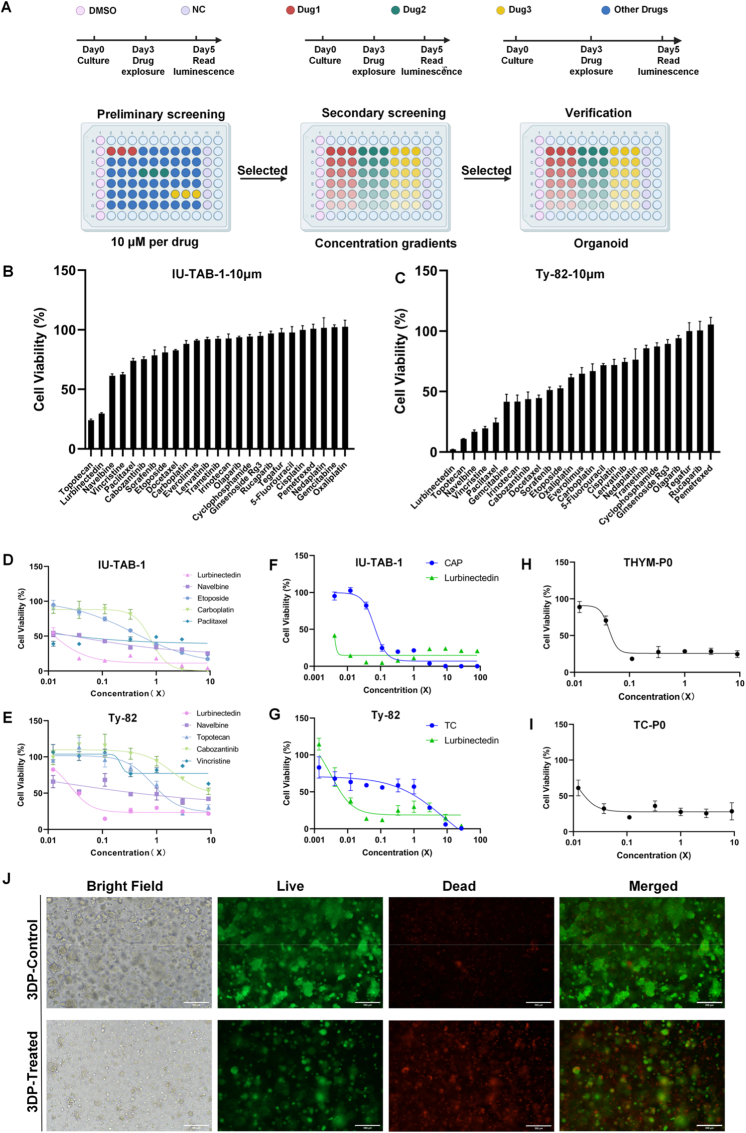
(A) Schematic of the multi-stage drug screening workflow including preliminary, secondary, and validation assays. (B–C) Cell viability of IU-TAB-1 cells and Ty-82 cells after treatment with 25 candidate drugs (10 μM) in 3D culture. (D–E) Dose-response curves of IU-TAB-1 cells (D) and Ty-82 (E) cells treated with top five effective compounds. (F–G) Comparison of lurbinectedin with standard chemotherapeutic agents in IU-TAB-1 cells (F) and Ty-82 cells(G). (H–I) Concentration-response curves of lurbinectedin in 3DP THYM (H) and TC (I) organoids. (J) Representative bright-field, live/dead and merged fluorescence images of 3DP organoids treated with lurbinectedin or vehicle control.Scale bars: 200 μm.

A two-stage high-throughput drug screening was conducted using bioprinted TET cell line models and corresponding patient-derived organoids to identify compounds with potent antitumor activity (Fig. 4A). In the primary screening, 25 candidate drugs at single dose of 10 μM were tested. In 3D THYM models generated from the IU-TAB-1 cell line (derived from a type AB thymoma sample), lurbinectedin exhibited the strongest inhibitory effect, reducing cell viability to below 50% and outperforming all other tested compounds (Fig. 4B). A similar trend was observed in 3D TC models derived from the Ty-82 cell line (derived from an undifferentiated thymic carcinoma sample) (Fig. 4C). Consistent findings were obtained in 2D cultures (Supplementary Fig. 5A–B)

The second-round screening further evaluated the top five compounds from the primary screen through concentration-gradient assays to determine their half-maximal inhibitory concentrations (IC_50_). Lurbinectedin showed a clear dose-dependent reduction in cell viability in both and Ty-82 cells and achieved the lowest IC_50_ values among all tested candidates (Fig. 4D and E). In addition, we compared the IC_50_ of lurbinectedin between 3DP models and traditional Matrigel models in drug screening assays. For Ty-82 cell line (Supplementary Fig. 5C–D), the corresponding values were 3.23 nM (3DP) and 3.42 nM (Matrigel). For the IU-TAB-1 cell line, the IC_50_ of lurbinectedin was 12.59 nM in the 3DP model versus 9.55 nM in the Matrigel model (Supplementary Fig. 5E–F). These results not only validate the anti-tumor efficacy of lurbinectedin via dual-model confirmation but also clearly demonstrate that the 3DP model possesses drug screening performance comparable to that of the conventional Matrigel model. Comparative assessment with first-line chemotherapeutic agents revealed that both thymic carcinoma and thymoma cell lines were more sensitive to lurbinectedin than to conventional drugs, including etoposide, carboplatin, and paclitaxel (Fig. 4F and G).

To further validate the antitumor efficacy under physiologically relevant conditions, lurbinectedin was tested in the bioprinted organoid model derived from patient tissue that mimics the native tumor microenvironment. Consistent with the cell line screening results, lurbinectedin markedly suppressed cell viability in both TC and THYM organoids (Fig. 4H and I). Morphologically, organoids without treatment exhibited regular spheroidal architecture with dense cellular aggregation, while lurbinectedin-treated organoids displayed reduced volume, disrupted boundaries, and extensive debris formation (Fig. 4J, Supplementary Fig. 5G–H). Live/dead staining further confirmed the cytotoxic effect: control organoids showed predominantly live cells, whereas treated organoids displayed substantial loss of viability with abundant dead cells. Collectively, these results verify that lurbinectedin effectively suppresses tumor growth and viability in both cell-line and organoid 3D models, validating its potential as a therapeutic agent.

### 2.5 Transcriptomic profiling reveals regulatory mechanisms of lurbinectedin in TC cells

**Fig. 5:**
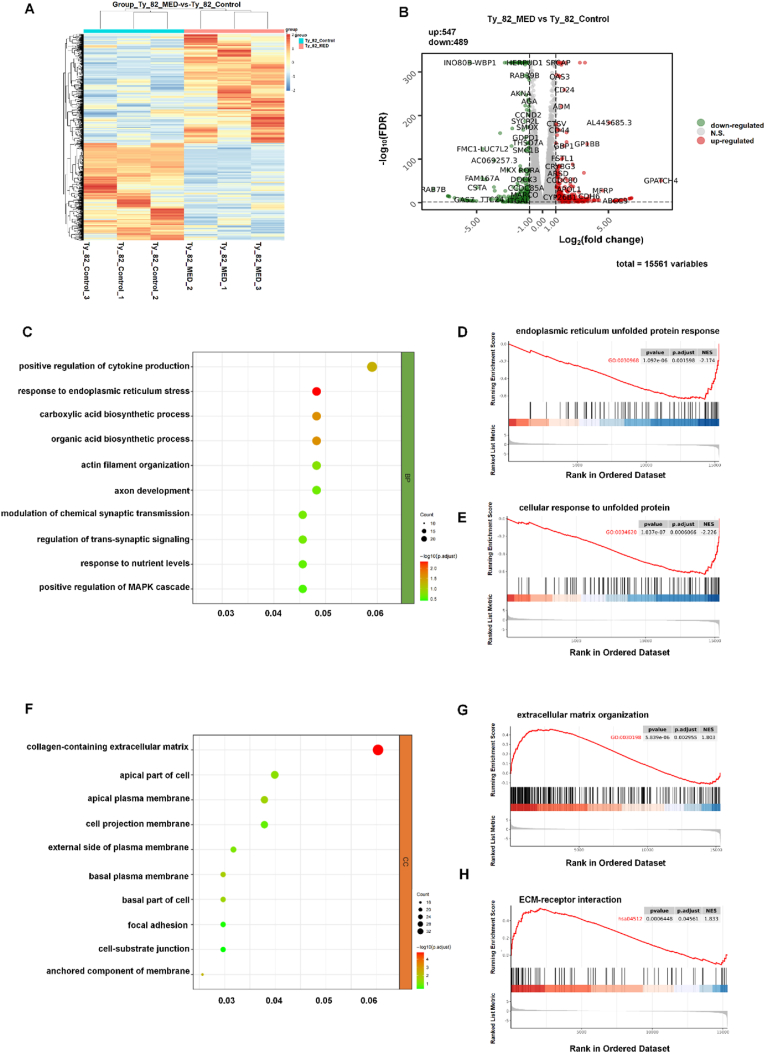
(A) Heatmap of gene-expression profiles of Ty-82 cells in the control and lurbinectedin-treated groups.(B) Volcano plot showing differentially expressed genes (DEGs) between treated and control groups.(C) Ty-82 GO downregulated pathways enrichment of Biological Process(BP).(D-E) Gene-set enrichment analysis (GSEA) for downregulated processes including the endoplasmic-reticulum unfolded-protein response and cellular response to unfolded protein.(F) Ty-82 GO upregulated pathways enrichment of Cellular Component(CC).(G-H) GSEA of extracellular-matrix organization and ECM-receptor interaction.

To elucidate the molecular mechanisms underlying the antitumor effects of lurbinectedin, RNA sequencing was performed on the 3D models of TC cell line Ty-82 before and after drug treatment. The heatmap in Fig. 5A shows distinct gene-expression patterns between the control and lurbinectedin-treated groups. Differential expression analysis identified 547 upregulated and 489 downregulated genes (Fig. 5B), indicating that lurbinectedin induces extensive transcriptional remodeling in TC cells.

Downregulated pathways are mainly concentrated in biological processes that maintain cell homeostasis, such as stress adaptation and cytokine regulation (Fig. 5C and Supplementary Fig. 6A–C). Fig. 5D demonstrates that the “endoplasmic reticulum unfolded protein response” pathway is significantly enriched in downregulated genes; Fig. 5E focuses on “cellular response to unfolded protein”, further verifying that lurbinectedin can inhibit the proteotoxic stress regulatory pathway in Ty-82 cells. These results indicate that lurbinectedin may disrupt cellular protein homeostasis and impair the cell's ability to cope with endoplasmic reticulum stress by downregulating stress-related pathways, laying the groundwork for inducing cell apoptosis.

Fig. 5F and Supplementary Fig. 6D–F shows that the core enriched terms are dominated by extracellular matrix (ECM)-related components, including “collagen-containing extracellular matrix” and “cell-substrate junction”. It's suggested that upregulated pathways focus on the structural components and dynamic organization of the ECM. Further, Fig. 5G demonstrates that this pathway is significantly activated. Fig. 5H further confirms that lurbinectedin enhances the adhesion and signal transduction between Ty-82 cells and the ECM. By upregulating ECM-related pathways, lurbinectedin reshapes the interaction between tumor cells and the microenvironment, which may affect the migration and invasion capabilities of tumor cells.

In the thymic carcinoma cell line Ty-82, lurbinectedin exerts a bidirectional synergistic antitumor regulatory effect. On one hand, it downregulates pathways linked to endoplasmic reticulum stress and protein homeostasis, disrupting cellular homeostasis to induce apoptosis. On the other hand, it upregulates pathways involved in ECM organization and receptor crosstalk, reshaping interactions between tumor cells and the microenvironment. From a transcriptomic perspective, these results clarify the molecular mechanism through which lurbinectedin regulates the survival and microenvironmental interactions of thymic cancer cells.

### 2.6 Lurbinectedin mediates transcriptional regulation in THYM cells

**Fig. 6:**
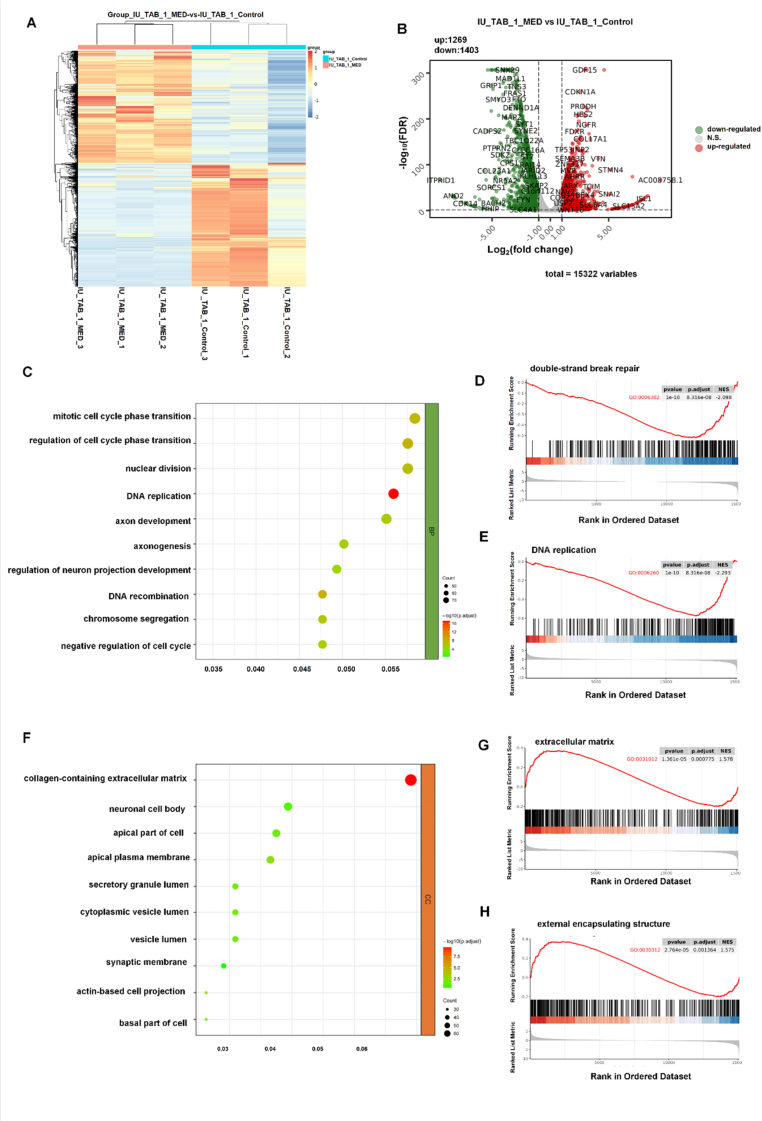
(A) Heatmap showing differential gene-expression profiles of IU-TAB-1cells in control and lurbinectedin-treated groups.(B) Volcano plot displaying up- and downregulated genes. (C) IU-TAB-1 GO downregulated pathways enrichment of Biological Process(BP).(D-E) Gene-set enrichment analysis (GSEA) for downregulated processes including the double-strand break repair and DNA replication.(F) IU-TAB-1 GO upregulated pathways enrichment of Cellular Component(CC).(G-H) GSEA of extracellular-matrix and external encapsulating structure.

To explore the molecular response of THYM cells to lurbinectedin, RNA sequencing was performed on the 3D models with IU-TAB-1 cell line before and after drug treatment. The heatmap (Fig. 6A) shows distinct clustering between the control and lurbinectedin-treated groups, while the volcano plot (Fig. 6B) demonstrates extensive transcriptional reprogramming, with 1269 genes upregulated and 1403 genes downregulated.

Down-regulated genes are mainly enriched in cell proliferation and DNA metabolism-related processes (Fig. 6C and Supplementary Fig. 7A–C), indicating that these pathways regulate cell cycle progression and DNA metabolism. GSEA results (Fig. 6D and E) further confirm that lurbinectedin down-regulates DNA replication-related pathways, interfering with key cell proliferation processes. Thus, lurbinectedin inhibits IU-TAB-1 cell proliferation by down-regulating cell cycle, DNA replication and repair pathways. To validate the transcriptional regulatory trend of lurbinectedin in inhibiting the DNA repair pathway, we performed immunofluorescence assays for γ-H2AX (a specific biomarker of DNA double-strand breaks) in both IU-TAB-1 and Ty-82 cell lines (cultured in a 3DP model). As shown in Supplementary Fig. 8A–B, both cell lines exhibited a consistent dose-dependent trend, with the γ-H2AX positivity rate increasing as the drug concentration was raised (the drug concentrations in the figures were increased in multiples of lurbinectedin Cmax).The concentration-dependent increase in γ-H2AX directly demonstrates that the drug induces the accumulation of DNA double-strand breaks (DSBs). However, it is worth noting that the absence of DNA repair-related entries in the enriched pathways. It may be attributed to the potential initiation of DNA repair at the protein activity level, or the intensity of its transcriptional regulation being lower than that of stress and inflammatory pathways, thus failing to be captured by enrichment analysis. In subsequent in-depth studies, this explanation can be further validated through targeted detection of repair genes/activity, cell cycle, and repair efficiency.

Up-regulated genes are predominantly enriched in extracellular matrix (ECM) and cellular structural components (Fig. 6F and Supplementary Fig. 7D–F), with the most significant being collagen-containing ECM. This suggests up-regulated pathways regulate the dynamic changes of ECM and cellular structures. GSEA results (Fig. 6G) confirm the ECM pathway is significantly activated; Fig. 6H further shows lurbinectedin up-regulates ECM-related structural regulatory processes. These results indicate that lurbinectedin reshapes the structural association between IU-TAB-1 cells and the microenvironment via up-regulating ECM-related pathways.

Lurbinectedin has two antitumor effects on IU-TAB-1 thymoma cells: proliferation inhibition and microenvironment remodeling. It directly suppresses cell proliferation by down-regulating cell cycle, DNA replication and repair pathways. Additionally, it reshapes cell-microenvironment interactions by up-regulating ECM-related pathways. At the transcriptomic level, this study clarifies how lurbinectedin regulates the biological behaviors of IU-TAB-1 cells.

### 2.7 Bioprinted 3D TET models enable identification and validation of biomarkers for lurbinectedin response

To identify potential efficacy-related biomarkers of lurbinectedin, we integrated transcriptomic analysis and experimental validation in THYM and TC cell 3D models.

**Fig. 7:**
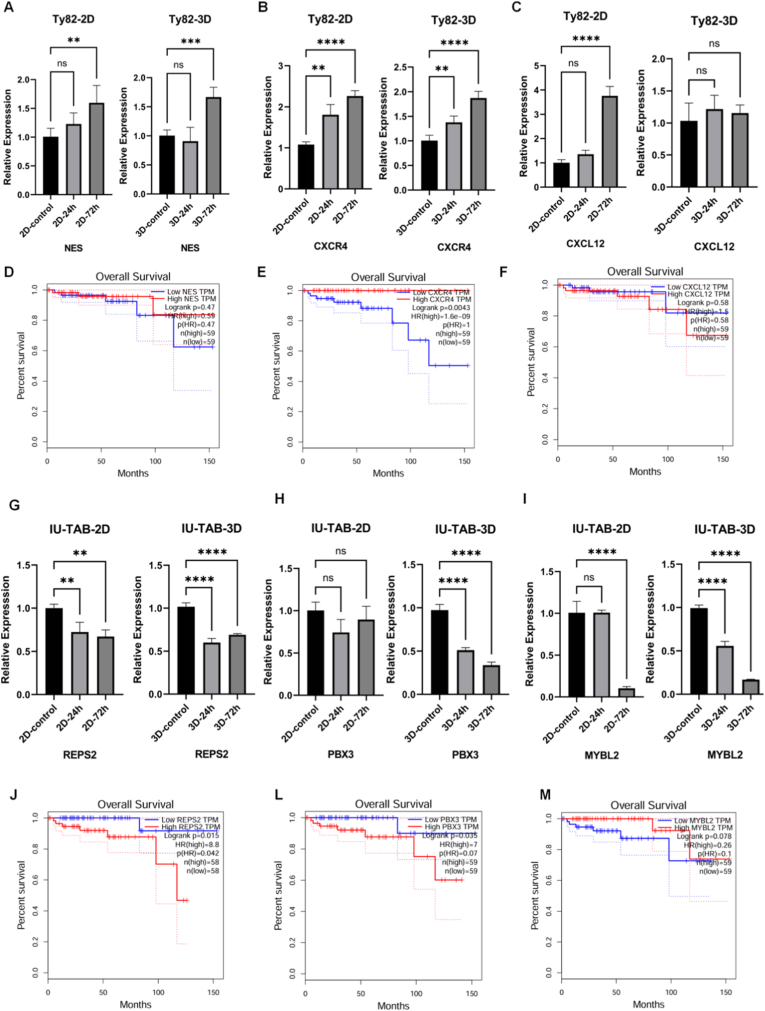
(A–C) Relative expression of NES, CXCR4, and CXCL12 in Ty-82 cells (2D and 3D) after lurbinectedin treatment.(D–F) Kaplan–Meier analysis of overall survival based on NES, CXCR4, and CXCL12 expression levels.(G–I) Relative expression of REPS2, PBX3, and MYBL2 in IU-TAB-1 cells (2D and 3D) after lurbinectedin treatment.(J–M) Kaplan–Meier curves showing overall survival of patients with high or low expression of REPS2, PBX3, and MYBL2.

In the TC cell line Ty-82, three significantly upregulated genes, CXCL12, CXCR4, and NES, were identified post-lurbinectedin treatment, and functional enrichment revealed their roles in immune microenvironment regulation and neural signaling [41,42]. Consistent with RNA-seq results, qRT-PCR confirmed significant upregulation of CXCR4 and NES in both 2D and 3D cultures (Fig. 7A and B), whereas CXCL12 exhibited culture-dependent expression (Fig. 7C). Increase of expression after treatment was significant in 2D cultures but not significant in 3D models. This observation aligns with prior reports on bone marrow microenvironment reconstruction, where 2D monolayer culture induced abnormal CXCL12 overexpression, while 3D hydrogel culture restored its physiological level by mimicking native matrix stiffness and adhesion signaling [43]. CXCL12 expression strictly depends on cell–ECM interactions [44], and therefore, the preservation of adhesion cues in 3D culture likely prevents artificial upregulation induced by the 2D environment. These findings indicate that 2D systems may yield false-positive results for chemokine expression due to the absence of biomechanical and adhesion feedback mechanisms.

Among the upregulated genes, CXCR4 was significantly associated with patient overall survival (Fig. 7D–F), consistent with pan-cancer evidence. A meta-analysis involving 1872 non–small cell lung cancer patients confirmed that high CXCR4 expression correlates with poor prognosis and serves as an independent predictor of metastasis [45]. Mechanistically, CXCR4 contributes to malignancy by activating PI3K/AKT signaling to enhance proliferation and metastasis [46] and by inducing immunosuppressive remodeling of the tumor microenvironment [47]. Given that TC progression and therapeutic response are tightly linked to immune dysfunction, elevated CXCR4 expression not only reflects immune dysregulation but also confers significant prognostic value, positioning it as a key predictive biomarker for lurbinectedin response and TC outcome.

In the THYM cell line IU-TAB-1, three significantly downregulated genes, MYBL2, PBX3, and REPS2, were identified from RNA-seq data before and after lurbinectedin treatment. Functional enrichment analysis indicated that these genes were mainly involved in cell-cycle progression, epigenetic regulation, and proliferation control [[48], [49], [50]]. Quantitative real-time PCR (qRT-PCR) confirmed the RNA-seq findings, revealing consistent downregulation across culture systems with varying degrees of sensitivity (Fig. 7G–I). Both MYBL2 and REPS2 were significantly reduced in 2D and 3D cultures, whereas PBX3 exhibited marked suppression exclusively under 3D culture conditions. Clinical correlation analysis showed that REPS2 and PBX3 expression levels were strongly associated with overall survival (Fig. 7J–L), while MYBL2 showed a weaker relationship (Fig. 7M). Notably, REPS2 demonstrated the most stable downregulation across systems and the strongest correlation with patient prognosis, highlighting its potential as a reliable biomarker for both lurbinectedin efficacy and THYM prognosis.

The exclusive downregulation of PBX3 in the 3D culture system underscores the importance of the microenvironment in gene regulation. Unlike 2D monolayers, which disrupt intercellular and stromal signaling, 3D culture preserves cell–cell and cell–matrix interactions, allowing lurbinectedin to act more effectively on microenvironment-dependent transcriptional pathways [51]. As a HOX coactivator, PBX3 requires intercellular contact cues for transcriptional modulation [52] hence, the structural and biochemical cues maintained in 3D culture likely facilitate more physiologically relevant suppression of this pathway. These findings emphasize that 3D bioprinted organoids not only provide a more accurate modeling platform for drug response but also enable context-specific biomarker discovery that cannot be captured in conventional 2D systems.

The study establishes the first proteomics-informed, 3D-bioprinted organoid platform for TETs, including TC and THYM, enabling precise modeling of the clinically relevant TET, high-throughput drug screening, and biomarker discovery. By integrating ECM characterization with photocurable bioink design, we achieved the first biophysically biomimetic reconstruction of the TET microenvironment that faithfully reproduced the histological architecture, genomic profile, and mechanical stiffness of primary TET tumors. Using this platform, lurbinectedin was identified as a potent therapeutic candidate that exerts significant regulatory effects on tumor stress responses and microenvironmental remodeling. Moreover, further analysis highlighted several genes as efficacy-associated biomarkers with strong prognostic potential, illustrating how 3D-printed tumor models can bridge drug-response evaluation and translational biomarker identification.

Compared with conventional Matrigel-based organoid systems, the 3D-bioprinted TET models offer superior reproducibility, structural controllability, and molecular fidelity. The proteomic mapping of decellularized TET tissues revealed collagen- and glycoprotein-dominant ECM features, guiding the rational selection of printing materials and achieving mechanical properties closely matching those of native tissues. This design allowed the models to retain subtype-specific genetic alterations, such as CNV patterns in TC and THYM, and to maintain intercellular and stromal signaling necessary for drug-response fidelity. Clinically, this platform addresses unmet needs for rare TETs with limited standard therapies. Personalized organoids can be built from biopsy tissues within 1–2 weeks enabling rapid drug sensitivity testing to guide optimal regimens. Identified biomarkers can be integrated into clinical workflows detecting them in biopsies pre-selects responsive patients reducing ineffective treatment and side effects. Mechanistically, the transcriptomic data demonstrate that lurbinectedin acts beyond canonical DNA damage to reshape transcriptional networks, integrating metabolic stress suppression with ECM and neural interaction reprogramming. These findings refine our understanding of how transcriptional inhibitors function in complex tumor systems and highlight the importance of modeling the tumor–stroma interface when evaluating mechanistically diverse drugs.

Despite these advantages current models have limitations for clinical translation. They lack key native tumor microenvironment components including immune cells that mediate TET immune escape functional vascular networks critical for simulating in vivo drug delivery and nutrient exchange and native thymic stromal cells whose paracrine signals regulate TET growth. Long-term passaging may also cause loss of subtype-specific molecular features undermining long-term drug testing stability. While these limitations call for targeted optimization in future research, the platform still delivers substantial translational advances for preclinical evaluation of rare thoracic malignancies. The 3D-printed model we developed addresses key limitations of traditional models through adjustable matrix stiffness, customizable structural design, and precise component ratios. For instance, it resolves batch-to-batch variability in Matrigel and morphological heterogeneity in organoids. We correlate these technological advantages with core challenges in studying rare tumors, including limited patient samples, low model reproducibility, and poor clinical translatability of conventional models.

From a translational perspective, this study provides both a methodological and conceptual advance for preclinical evaluation of rare thoracic malignancies. The 3D-bioprinted system establishes a reproducible, human-relevant framework for linking molecular mechanisms to therapeutic outcomes. Importantly, the differential responses observed between 2D and 3D cultures underscore the need for microenvironment-aware strategies in biomarker discovery. Future studies may expand this platform toward multi-cell co-culture systems incorporating immune or vascular components to capture intercellular dynamics under pharmacological perturbation. Together, these advances position 3D-bioprinted TET organoids as a powerful tool for precision oncology, enabling predictive biomarker identification and rational drug development for clinically underserved tumor types.

## 3 Methods and materials

### 3.1 Human specimens and ethical approval

Tumor tissues and adjacent normal tissues were collected from TETs patients in the Department of Thoracic Surgery at Shanghai Chest Hospital. Informed consent was obtained from all participants, and the study was approved by the Ethics Committee of Shanghai Chest Hospital (No. KS25049).

### 3.2 Tumor decellularization

First, seven tumor samples stored at −80 °C were subjected to three cycles of freeze–thaw treatment. Afterwards, the tissues were rinsed repeatedly with deionized water until no visible blood coloration remained. Subsequently, the samples were incubated with Sodium Dodecyl Sulfonate (SDS, 1% w/v,Titan) under magnetic stirring at 300 rpm for 2h at 4 °C. After that, SDS was removed by thorough washing with deionized water. Next, the tissues were treated with 1M NaCl(Titan) solution under magnetic stirring at 300 rpm for 30 min at 4 °C. After treatment, the samples were rinsed again with deionized water to eliminate salt residues. In the end, the tissues were further stirred in deionized water at 300 rpm for 1h at 4 °C.

### 3.3 Proteomics analysis

Seven samples were used for proteomics (Table 2). Proteomic analysis was performed by Shanghai OE Biotech Co., Ltd. At first, total proteins were extracted from each sample following standard protocols and quantified using the Bicinchoninic acid (BCA) protein concentration assay (Thermo Fisher Scientific). According to the measured protein concentration, appropriate amounts of protein from each sample were diluted with lysis buffer to equal concentrations and volumes. SP3 magnetic beads were added to the protein solution, followed by the addition of 100% acetonitrile (ACN, Thermo Fisher Scientific). The mixture was incubated at room temperature for 20 min. After magnetic separation, the supernatant was removed, and the beads were sequentially washed twice with 70% ethanol and twice with 70% ACN. The beads were then resuspended in 50 mM ammonium bicarbonate (ABC, Yuanye) solution. Reduction was performed by adding dithiothreitol (DTT, Adamas-beta) and incubating at 55 °C for 30 min, followed by alkylation with chloroacetamide (CAA, Shenggong). Proteins were digested with sequencing-grade trypsin at 37 °C with shaking at 1500 rpm. Digested peptides were desalted using a SOLA™ SPE 96-well plate (Thermo Fisher Scientific). The cartridge was activated with 200 μL methanol (CNW) three times and equilibrated with 200 μL of 0.1% formic acid (CNW) in water three times. Samples (50–500 μL) were loaded twice under vacuum with a controlled flow rate of 1 mL/min (approximately one drop per second). The cartridge was washed three times with 200 μL of 0.1% formic acid in water. Peptides were eluted three times with 150 μL of 50% acetonitrile in water containing 0.1% formic acid, yielding a total of 450 μL eluate, which was dried under vacuum. Then peptides were separated on a C18 reversed-phase analytical column using a Vanquish Neo UHPLC (ThermoFisher) with a flow rate of 700 nL/min. Subsequently, protein quantification was performed using the Data Independent Acquisition (DIA) approach. Raw Mass Spectrometry (MS) data were processed using theoretical spectra from the Fasta database. Protein identification was filtered with a false discovery rate (FDR) which is 0.01. Functional enrichment analyses, including Gene Ontology (GO) annotation and Kyoto Encyclopedia of Genes and Genomes (KEGG) pathway enrichment, were performed subsequently.

### 3.4 Tissue dissociation and single cell Isolation

Human thymic carcinoma tissue were kept in MACS Tissue Storage Solution for less than 24 h before experiment processing. The fresh tissues were washed with cold antibiotic-supplemented Hank's solution for three times following. Mechanical dissociation was performed in centrifuge tubes containing small amount of pre-warmed enzyme solution. The enzyme solution is consisted of Hank's with 1.5 mg/ml Collagenase Types Ⅳ, 1 mg/ml hyaluronidase, 0.1 mg/ml DNase I, and 1% PSA. The specimen was minced into small fragments at about 1-2 mm3 using sterile surgical scalpels. The digested solution was transferred to the T-25 flask, and the suspension was digested and placed in the cell culture incubator under constant agitation for 30 min. The digestion process was stopped by adding a double volume of dissociation stop buffer. The cell suspension was filtered through sterile 100 μm cell strainer. The cells suspension was centrifuged at 400×*g* for 5 min at 4 °C. The supernatant was carefully aspirated.

### 3.5 Cell lines and cell culture

The Ty-82 cell line, derived from an undifferentiated thymic carcinoma sample (JCRB1330), was purchased from Zhejiang Meisen Cell Technology Co., Ltd. The IU-tab-1 cell line, derived from a type AB thymoma sample, was acquired from the Applied Biological Materials Inc. (Abm, BC, Canada). Ty-82 cells are cultured in RPMI 1640 medium (Abm, BC, Canada) contained 20% fetal bovine serum (FBS; Gibco) and 1% penicillin-streptomycin (Gibco). IU-tab-1 cells are maintained in RPMI 1640 medium (Abm, BC, Canada) contained 10% fetal bovine serum (FBS; Gibco) and 1% penicillin-streptomycin (Gibco). All cellular cultures were upheld in a humidified incubation chamber with 5% CO_2_ at 37 °C and regularly tested for mycoplasma contamination.

### 3.6 Establishment of 3D TET patient-derived tissue (PDT) models

Single-cell suspension was centrifuged at 400×*g* for 5 min, and the cell pellet were resuspended in a small amount volume of advanced DMEM/F-12. To maintain cell viability, the cell suspension was kept on ice before bioprinting. Hybrid gelatin methacryloyl (GelMA) and hyaluronic acid methacryloyl (HAMA) were synthesized and provided by Yuju Technology, Shanghai, China. The photoinitiator lithium phenyl-2,4,6-trimethylbenzoylphos phinate (LAP) was obtained from EngineeringForLife. Hydrogel precursor solution was prepared by dissolving GelMA, HAMA, and LAP in DPBS at a concentration of 10% (w/v),10% (w/v), 2% (w/v) separately, and then filter sterilized through 0.22 μm syringe filters. The construction of 3D TET model was performed using a DLP-based 3D bioprinter (Cyberiad Biotech, Shanghai, China). Cells suspension were resuspended in the hydrogel precursor solution and the final concentration of GelMA, HAMA, and LAP inside the bioink were adjusted to 4%, 0.5%, and 0.1%. The mixture was gently homogenized by pipetting to ensure a uniform cell distribution while minimizing bubble formation. To control the size and the shape of the samples, the 3D bioprinting model was constructed using the Azure series light-curing bioprinter developed by Saifol Biotechnology. The key printing parameters adopted in this experiment were as follows: the exposure intensity was adjusted to 35%, the curing exposure time was set at 23s, and the printing thickness was configured at 0.5 mm to ensure the accuracy and biocompatibility of the printed structure. The bioink was pipetted into a 24-well plate followed by the initiation of bioprinting. The printed sample was gently rinsed with DPBS and maintained in 1 ml of complete culture media for the entire length of the experiment. It was subsequently incubated at 37 °C and 5% CO2 for further culture and analysis. Complete cell culture contain Advanced DMEM/F-12 supplemented with 50 ng ml-1 EGF, 20 ng ml-1 bFGF, 20 ng ml-1 FGF-10, 50 ng ml-1 Wnt-3A, 200 ng ml-1 R-spondin-1, 100 ng ml-1 Noggin, 10 ng ml-1 IGF-1, 10 ng ml-1 IL-2, 500 ng ml-1 A83-01, 1X B-27, 10 μM Y-27632, 200 mM glutamax, 100 U ml-1 penicillin and 100 μg ml-1 of streptomycin(Table 3). The media was changed three times a week.

### 3.7 Immunofluorescence (IF) staining

3D bioprinted thymic carcinoma PDT Models in well plate were fixed with 4% paraformaldehyde (PFA) at 4 °C for overnight. After fixation, PFA was removed, and the models were washed three times with DPBS. Following the fixation, permeabilization was performed using 0.3% Triton X-100 in DPBS at room temperature under gentle agitation for 30 min. Non-specific binding sites were blocked by incubating the samples in the blocking buffer for 2 h at room temperature.Without intermediate washing, the samples were incubated with primary antibodies diluted in the blocking buffer overnight at 4 °C. The following primary antibodies were used: Pan-CK (#MA513203, invitrogen) at a dilution of 1:100, CD117 (AF1356-SP, R&D system) at a dilution of 1:100, CD56/NACM1 (#ab313779, abcam) at a dilution of 1:100, p63 (#ab124762, abcam) at a dilution of 1:100, TdT (#TB4932S, abmart) at a dilution of 1:100, and Anti-gamma H2A.X (phospho S139) antibody (#ab2893, abcam) at a dilution of 1:100. Following primary antibody incubation, the samples were washed with DPBS. Subsequently, the samples were incubated with species-specific secondary antibodies conjugated to Alexa Fluor 488, Alexa Fluor 594, Alexa Fluor 647 (1:200, abcam) diluted in the blocking buffer. This secondary antibody incubation was carried out for 4 h at room temperature in the dark. Following secondary antibody washes, the samples were incubated with DAPI staining solution (Beyotime) for 10 min at room temperature in the dark. For imaging, individual PDT models were carefully extracted from cell plate and transferred onto the confocal imaging dish. Image acquisition was performed using an Olympus spinSR10 super-resolution spinning disk confocal microscope.

### 3.8 Flow cytometry assay

TETs organoids were digested into single cells using 3DP digestive enzymes and TrypLE Express, then resuspended for subsequent experiments. The cells were incubated at room temperature for 10-15 min with FC-block (130-091-935,Miltenyi Biotec) to reduce nonspecific staining. Fluorescently labeled 7AAD (420404,Biolegend), CD45 (368512,Biolegend), CD326 (369816,Biolegend),CD8 (344704,Biolegend) and CD3 (317342,Biolegend) antibodiewere added sequentially, followed by 30-min incubation in the dark. The cells were washed twice with flow cytometry buffer to remove unbound antibodies. Data were detected using the BD LSRFortessa instrument and analyzed with FlowJo software.

### 3.9 Drug screening in 2D and 3D models

Given the limited clinical treatment options for TETs and the current clinical guidelines that prioritize chemotherapeutic agents as first-line therapy [5], we will screen two categories of drugs: 1) classic chemotherapy widely used in common solid tumors; 2) Multi-target inhibitors, (e.g.,cabozantinib [53] and Lenvatinib [54]). Additionally, we have developed corresponding drug screening tables in Table 4. The chemical library was obtained from TargetMol. The detection reagent CellTiter-Glo (CTG) was acquired from Promega. For the primary drug screening, both two-dimensional (2D) and three-dimensional (3D) models were used to compare drug efficacy across different cellular contexts. Prior to screening, the cell lines were resuscitated and expanded. Cells were seeded in 96-well at a density of 20000 per well. The seeded plates were incubated for 72 h. Following incubation, each chemical compounds was diluted and transferred to the assay plates, resulting in a final test concentration of 10 μM and a final DMSO concentration of less than 0.2%. The positive control (cell culture medium) were included on each plate. After 48 h of compounds exposure, cell viability was quantified using CTG according to the manufacturer's protocol. The luminescence was measured and recorded using a microplate reader. The percentage of cell viability was normalized to the control group. Compounds with an viability rate less than 50% as hit compounds for further validation.

Based on the primary drug screening results, a dose-response assay was performed. The top 5 hit compounds with the lowest viability rates for each cell line were selected. The baseline concentration for IC_50_ testing was set as 9 × the Cmax of each compound. Each concentration and control was tested in at least four replicates. The PDT model (2 μl per well) was inoculated in 96-well plates, with compound concentrations and dosages matching those used in the primary drug screening protocol. Luminescence intensity after medication was measured with CTG using the same microplate reader and settings as primary drug screening experiment to ensure the data consistency. Dose-response curve with responded to each compound was plotted in GraphPad Prism and the IC_50_ values were calculated via non-linear regression analysis. Based on the monotherapy result, the efficacy of the most sensitive compound with lowest IC_50_ values (Lurbinectedin) for both cell lines was compared to the clinical combination regimen.

To validate the findings from the established cell lines in a more clinically relevant model, drug sensitivity testing was extended to patient-derived primary cells. The primary cells were cultured as 3D PDT models same as previously, and treated with a gradient of concentrations of Lurbinectedin. After 48 h of drug exposure, cell viability was quantified using the highly sensitive CellTiter-Glo 3D Assay.

### 3.10 RNA sequencing (RNAseq)

TET cells were cultured in a 3D bioprinted model for 48 h, incubated with Lurbinectedin for 24 h, then collected. Cells were released by digesting hydrogels with digestive enzyme working solution at 37 °C for 10 min, centrifuged at 400g for 5 min, supernatants discarded, and pellets resuspended in 1 ml Trizol (Thermo Fisher) for RNA extraction.

Post-extraction, RNA integrity was assessed via 1.5% agarose gel electrophoresis or fragment analyzer; concentration and purity were measured with NanoDrop. mRNA was enriched using Oligo (dT)-conjugated magnetic beads.

Transcriptome libraries were constructed per VAHTS Universal V8 RNA-seq Library Prep Kit (Illumina) protocol: target RNA was fragmented with ion fragmentation reagent and bound to random primers, followed by first-strand reverse transcription to form RNA-cDNA hybrids; second-strand synthesis reagents cleaved hybrid RNA (remaining RNA primed second-strand cDNA synthesis); second-strand cDNA underwent end repair, A-tailing, and ligation with "Y"-shaped adapters; adapter dimers were removed via magnetic bead selection, library templates enriched by PCR, and libraries recovered with magnetic beads.

Libraries were quantified with Qubit 4.0 (Thermo Fisher Scientific, USA) and sequenced on Illumina NovaSeq (PE150).

### 3.11 Whole exome sequencing (WES)

Eight samples (tumor tissues from TC and TYHM patients, adjacent non-tumor tissues, 3D-printed organoids, and Matrigel-grown organoids) underwent whole exome sequencing (WES) by Shanghai Ouya Biotechnology Co., Ltd. Genomic DNA was extracted, qualified by electrophoresis, and used for library construction: qualified DNA was fragmented to 150–220 bp via Covaris, followed by library construction and capture with the Agilent SureSelect Human All Exon V6 kit. Qualified libraries were paired-end sequenced.Raw sequencing data were processed via bioinformatics analysis.Raw data were quality-controlled with fastp to obtain clean reads using the following criteria.

SNPs and InDels were detected using GATK4's Haplotypecaller, with prior base quality recalibration (GATK4's BaseRecalibrator) against known SNP/InDel databases. Variants were filtered by QD ≥ 2.0 (retaining high-confidence variants) and annotated to Refseq, 1000 Genomes, EXAC, esp6500, gnomAD, SIFT, ClinVar, PolyPhen, MutationTaster, COSMIC, GWAS Catalog, and OMIM via Annovar.

Copy number variations (CNVs; gains/losses of genomic segments, a key structural variation subtype including deletions/duplications) in paired normal-tumor samples were detected using Control-FREEC.

### 3.12 Quantitative real-time polymerase chain reaction (qRT-PCR) analysis

Total RNA from TET cells was extracted using TRIzol reagent (Thermo Scientific, MA, USA). cDNA was reverse-transcribed and synthesized by NuovaGen HiScript III 1st Strand cDNA Synthesis Kit (R312-01/02, China).Perform quantitative detection using the NuovaGen ChamQ Universal SYBR qPCR Master Mix (Q711-02/03, China) on a real-time PCR instrument. Gene expression levels were computed using the 2^(-ΔΔCt) method. The primer sequences for MYBL2, PBX3, REPS2, CXCL12, CXCR4, NES,GAPDH are provided in Table 5.

### 3.13 Statistical analysis

The data from drug sensitivity testing were repeated 2-3 times and analyzed using GraphPad Prism software. The IC_50_ values of drug effects on cells were derived from nonlinear regression analysis of the data obtained by CellTiter-Glo 3D assay. Significance analysis was performed using one-way ANOVA, with P < 0.05 considered statistically significant. Detailed analysis descriptions were included in corresponding figure captions.

## CRediT authorship contribution statement

**Beibei Liu:** Methodology, Resources, Software, Validation, Visualization, Writing – original draft, Writing – review & editing. **Huiyan Cheng:** Investigation, Methodology, Validation, Visualization, Writing – original draft, Writing – review & editing. **Keke Yu:** Methodology, Project administration, Resources, Validation, Visualization. **Wen Xu:** Methodology, Visualization, Writing – original draft, Writing – review & editing. **Xiaoting Tian:** Software, Supervision, Validation, Visualization, Writing – original draft. **Yuhan Xu:** Software, Validation, Visualization. **Yanbin Kuang:** Resources, Supervision, Validation. **Jun Lu:** Formal analysis, Investigation, Software, Supervision. **Rong Li:** Conceptualization, Project administration, Supervision. **Xiao Zhang:** Conceptualization, Methodology, Project administration. **Min Tang:** Conceptualization, Methodology, Resources, Supervision. **Jianxin Xue:** Conceptualization, Funding acquisition, Methodology, Project administration, Supervision. **Yuqing Lou:** Conceptualization, Funding acquisition, Methodology, Project administration, Resources, Supervision.

## Declaration of competing interest

The authors declare that they have no known competing financial interests or personal relationships that could have appeared to influence the work reported in this paper.

## Data Availability

Data will be made available on request.
