# Supplementary material for: Biomimetic 3D-Bioprinted organoids of thymic epithelial tumors for translational drug screening and biomarker identification
Source: Mater Today Bio. 2026 Feb 11;37:102878. doi: 10.1016/j.mtbio.2026.102878 (PMC12924735; doi:10.1016/j.mtbio.2026.102878)
Supplement: Multimedia component 1 [file mmc1.docx]

**Tables**

|  | **Pathological type** | **IHC** |
| --- | --- | --- |
| TC | High-grade neuroendocrine carcinoma | CK(+)，P63(-)，TdT(-)，CD5(-)，CD3(-)，CD20(-)，CD117(+)，CD56(+)，TTF-1(+)，SYN(+)，Ki-67(90%+) |
| THYM | Type A thymoma | CK(+)，P63(+)，TdT(Lymphocytes few-)，CD5(-)，CD117(-) |

**Table 1.** Pathological diagnosis of tumor tissue of patients

| **Patient** | **Pathological type** | **Sex** | **Age** |
| --- | --- | --- | --- |
| 01 | A | Male | 79 |
| 02 | A | Female | 69 |
| 03 | A | Female | 65 |
| 04 | AB | Male | 75 |
| 05 | AB | Female | 44 |
| 06 | B2 | Male | 66 |
| 07 | C | Female | 70 |

**Table 2.** The Baseline Characteristics of Patients

| Factors | Concentration | Roles in organoid culture |
| --- | --- | --- |
| EGF | 50 ng·ml^-^¹ | Activates the EGFR signaling pathway, promotes proliferationof organoid cells, and enhances intercellular adhesion |
| bFGF | 20 ng·ml^-^¹ | Regulates the cell cycle process, accelerates the proliferation of organoid cells and maintains stem cell characteristics |
| FGF-10 | 20 ng·ml^-^¹ | Specifically promotes the proliferation and self-renewal of epithelial stem cells. |
| Wnt-3A | 50 ng·ml^-^¹ | Activates the canonical Wnt/β-catenin pathway, maintains the stability of the organoid stem cell pool, drives stem cell proliferation. |
| R-spondin-1 | 200 ng·ml^-^¹ | Synergizes with the Wnt signaling pathway, enhances the efficiency of Wnt ligands, further promotes the proliferation of organoid stem cells. |
| Noggin | 100 ng·ml^-^¹ | Noggin supports the formation and functional maintenance of organoids by regulating BMP (Bone Morphogenetic Protein) signaling, either by maintaining the undifferentiated state of stem cells or by promoting the differentiation of specific cell types. |
| IGF-1 | 10 ng·ml^-^¹ | Activates the PI3K/Akt signaling pathway, promotes the metabolism and proliferation of organoid cells, inhibits apoptosis. |
| IL-2 | 10 ng·ml^-^¹ | Regulates the function of immune cells in the organoid microenvironment, indirectly supports the proliferation of organoid cells through the paracrine effect of immune cells. |
| A83-01 | 500 ng·ml^-^¹ | Specifically inhibits TGF-β receptor I , blocks the TGF-β-mediated cell growth inhibitory pathway, promotes the proliferation of organoid cells. |
| B-27 | 1X | Provides various nutrients and growth auxiliary factors required for the growth of organoid cells, maintains cell viability. |
| Y-27632 | 10 μM | Inhibits ROCK kinase activity, reduces organoid cell apoptosis, improves cell survival status. |
| Glutamax | 200 mM | Provides key energy substances and nitrogen sources for organoid cell proliferation. |

**Table 3.** Factors in organoids culture medium and their roles in organoid proliferation

|  | **Chemotherapy** | **Mechanism** | **Clinical Indications** |
| --- | --- | --- | --- |
| 1 | Cisplatin | Platinum-based alkylating agent which disrupt the double-stranded structure of DNA | Recommended as first-line chemotherapy drug for TET by NCCN guidelines^1^ |
| 2 | Carboplatin | Platinum-based alkylating agent which causing DNA double-strand damage | Alternative option (in combination with paclitaxel) for TET patients intolerant to cisplatin^1^ |
| 3 | Nedaplatin | Cisplatin analogs, DNA crosslinkers | Alternative options for TET patients intolerant to cisplatin/carboplatin^1^ |
| 4 | Paclitaxel | Paclitaxel class, inhibits microtubule depolymerization and blocks the G2/M phase of the cell cycle | The NCCN guidelines recommend carboplatin as a first-line chemotherapy regimen for TETs^1^ |
| 5 | Docetaxel | Paclitaxel, microtubule inhibitors | Second-line chemotherapy regimens for advanced/refractory TETs^1^ |
| 6 | Vinorelbine | Inhibit microtubule polymerization and block the cell cycle in the M phase | Non-surgical resectable locally advanced or metastatic non-small cell lung cancer, and monotherapy or combination chemotherapy for metastatic breast cancer^2,3^ |
| 7 | Vincristine | Vinca alkaloid class, microtubule inhibitors | head and neck tumors, lung cancer^4,5^ |
| 8 | Etoposide | Topoisomerase II inhibitor, disrupting DNA replication fork | Second-line chemotherapy for thymic carcinoma^1^ |
| 9 | Irinotecan | Topoisomerase I inhibitor, induces single-strand DNA breaks | Colorectal cancer, pancreatic cancer, small cell lung cancer^6,7,8^etc. |
| 10 | Topotecan | Topoisomerase I inhibitor, inhibiting DNA repair | Small cell lung cancer, advanced metastatic ovarian cancer^9,10^ |
| 11 | Gemcitabine | Antimetabolites, inhibiting DNA synthesis (pyrimidine analogs) | Second-line chemotherapy for thymic carcinoma^1^, lung cancer^11^ |
| 12 | Pemetrexed | Inhibits folate metabolism and disrupts DNA synthesis and repair in cancer cells | Other recommended second-line chemotherapy drugs for thymoma^1^, non-small cell lung cancer^12^ |
| 13 | Tegafur | Fluorouracil precursors | Rectal cancer^13^, Head and Neck Cancer^14^,etc. |
| 14 | Lurbinectedin | Topoisomerase II inhibitor, disrupts DNA structure | Small Cell Lung Cancer^15^ |
| 15 | Cyclophosphamide | Alkylating agents, which disrupt DNA structure | Lymphoma, Multiple Myeloma, Ovarian Cancer,etc^16^. |
| 16 | 5-Fluorouracil | Pyrimidine analogues, which inhibit the synthesis of DNA and RNA | Colorectal Cancer^17^  Head and Neck cancer^18^ |
| 17 | Oxaliplatin | Inhibiting the replication and transcription of cancer cell DNA | Pancreatic cancer^19^; Gastric cancer^20^ |
| 18 | Cabozantinib | Multi-target tyrosine kinase inhibitor (VEGFR, MET, RET, etc.) | Thyroid carcinoma^21^; Renal cell carcinoma^22^,etc. |
| 19 | Lenvatinib | Multi-target inhibitor (VEGFR1-3, FGFR, etc.) | Thyroid cancer^23^;Hepatocellular carcinoma^24^ |
| 20 | Sorafenib | Multi-target inhibitor (VEGFR、RAF、PDGFR.etc.) | Thyroid cancer^25^ ;Advanced renal cell carcinoma^26^ |
| 21 | Everolimus | mTOR inhibitors | Thymic carcinoma as one of the second-line chemotherapy options^1^ |
| 22 | Trametinib | MEK inhibitors | Ovarian cancer^27^；Non-small cell lung cancer^28^ |
| 23 | Olaparib | PARP inhibitors | Advanced ovarian cancer^29^;Breast Cancer^30^ |
| 24 | Rucaparib | PARP inhibitors (PARP1/2/3 inhibitors) | Ovarian Cancer^31^; Prostate cancer^32^, etc. |
| 25 | Ginsenoside Rg3 | Natural products capable of inhibiting angiogenesis and inducing apoptosis^33,34^ | Combined with chemotherapy for primary lung cancer and liver cancer (to enhance therapeutic efficacy and mitigate adverse chemotherapy reactions) |

**Table 4.** List of drugs for initial screening

Reference of **Table 4**:

1. Riely GJ, Wood DE, Loo BW, et al. Thymomas and Thymic Carcinomas, Version 2.2025, NCCN Clinical Practice Guidelines In Oncology. J Natl Compr Canc Netw. 2025;23(6):255-269. doi:10.6004/jnccn.2025.0027
2. Baggstrom MQ, Stinchcombe TE, Fried DB, Poole C, Hensing TA, Socinski MA. Third-generation chemotherapy agents in the treatment of advanced non-small cell lung cancer: a meta-analysis. J Thorac Oncol. 2007;2(9):845-853. doi:10.1097/JTO.0b013e31814617a2
3. Chan A, Verrill M. Capecitabine and vinorelbine in metastatic breast cancer. Eur J Cancer. 2009;45(13):2253-2265. doi:10.1016/j.ejca.2009.04.031
4. Clavel M, Vermorken JB, Cognetti F, et al. Randomized comparison of cisplatin, methotrexate, bleomycin and vincristine (CABO) versus cisplatin and 5-fluorouracil (CF) versus cisplatin (C) in recurrent or metastatic squamous cell carcinoma of the head and neck. A phase III study of the EORTC Head and Neck Cancer Cooperative Group. Ann Oncol. 1994;5(6):521-526. doi:10.1093/oxfordjournals.annonc.a058906
5. Zhang Y, Yang SH, Guo XL. New insights into Vinca alkaloids resistance mechanism and circumvention in lung cancer. Biomed Pharmacother. 2017;96:659-666. doi:10.1016/j.biopha.2017.10.041
6. Kciuk M, Marciniak B, Kontek R. Irinotecan-Still an Important Player in Cancer Chemotherapy: A Comprehensive Overview. Int J Mol Sci. 2020;21(14):4919. Published 2020 Jul 12. doi:10.3390/ijms21144919
7. Douillard JY, Sobrero A, Carnaghi C, et al. Metastatic colorectal cancer: integrating irinotecan into combination and sequential chemotherapy. Ann Oncol. 2003;14 Suppl 2:ii7-ii12. doi:10.1093/annonc/mdg723
8. Kipps E, Young K, Starling N. Liposomal irinotecan in gemcitabine-refractory metastatic pancreatic cancer: efficacy, safety and place in therapy. Ther Adv Med Oncol. 2017;9(3):159-170. doi:10.1177/1758834016688816
9. Hartwell D, Jones J, Loveman E, Harris P, Clegg A, Bird A. Topotecan for relapsed small cell lung cancer: a systematic review and economic evaluation. Cancer Treat Rev. 2011;37(3):242-249. doi:10.1016/j.ctrv.2010.07.005
10. Lihua P, Chen XY, Wu TX. Topotecan for ovarian cancer. Cochrane Database Syst Rev. 2008;2008(2):CD005589. Published 2008 Apr 16. doi:10.1002/14651858.CD005589.pub2
11. Baggstrom MQ, Stinchcombe TE, Fried DB, Poole C, Hensing TA, Socinski MA. Third-generation chemotherapy agents in the treatment of advanced non-small cell lung cancer: a meta-analysis. J Thorac Oncol. 2007;2(9):845-853. doi:10.1097/JTO.0b013e31814617a2
12. Pérez-Moreno MA, Galván-Banqueri M, Flores-Moreno S, Villalba-Moreno A, Cotrina-Luque J, Bautista-Paloma FJ. Systematic review of efficacy and safety of pemetrexed in non-small-cell-lung cancer. Int J Clin Pharm. 2014;36(3):476-487. doi:10.1007/s11096-014-9920-2
13. Casado E, Pfeiffer P, Feliu J, González-Barón M, Vestermark L, Jensen HA. UFT (tegafur-uracil) in rectal cancer. Ann Oncol. 2008;19(8):1371-1378. doi:10.1093/annonc/mdn067
14. Lee HL, Chen PH, Huang TC, et al. Tegafur-Uracil Maintenance Therapy in Non-Metastatic Head and Neck Cancer: An Exploratory Systematic Review. Curr Oncol. 2025;32(5):286. Published 2025 May 20. doi:10.3390/curroncol32050286
15. Paz-Ares L, Borghaei H, Liu SV, et al. Efficacy and safety of first-line maintenance therapy with lurbinectedin plus atezolizumab in extensive-stage small-cell lung cancer (IMforte): a randomised, multicentre, open-label, phase 3 trial. Lancet. 2025;405(10495):2129-2143. doi:10.1016/S0140-6736(25)01011-6
16. Emadi A, Jones RJ, Brodsky RA. Cyclophosphamide and cancer: golden anniversary. Nat Rev Clin Oncol. 2009;6(11):638-647. doi:10.1038/nrclinonc.2009.146
17. Vodenkova S, Buchler T, Cervena K, Veskrnova V, Vodicka P, Vymetalkova V. 5-fluorouracil and other fluoropyrimidines in colorectal cancer: Past, present and future. Pharmacol Ther. 2020;206:107447. doi:10.1016/j.pharmthera.2019.107447
18. Vermorken JB, Remenar E, van Herpen C, et al. Cisplatin, fluorouracil, and docetaxel in unresectable head and neck cancer. N Engl J Med. 2007;357(17):1695-1704. doi:10.1056/NEJMoa071028
19. Kamisawa T, Wood LD, Itoi T, Takaori K. Pancreatic cancer. Lancet. 2016;388(10039):73-85. doi:10.1016/S0140-6736(16)00141-0
20. Yamada Y, Higuchi K, Nishikawa K, et al. Phase III study comparing oxaliplatin plus S-1 with cisplatin plus S-1 in chemotherapy-naïve patients with advanced gastric cancer. Ann Oncol. 2015;26(1):141-148. doi:10.1093/annonc/mdu472
21. Elisei R, Schlumberger MJ, Müller SP, et al. Cabozantinib in progressive medullary thyroid cancer. J Clin Oncol. 2013;31(29):3639-3646. doi:10.1200/JCO.2012.48.4659
22. Abdelaziz A, Vaishampayan U. Cabozantinib for Renal Cell Carcinoma: Current and Future Paradigms. Curr Treat Options Oncol. 2017;18(3):18. doi:10.1007/s11864-017-0444-6
23. Cabanillas ME, Habra MA. Lenvatinib: Role in thyroid cancer and other solid tumors. Cancer Treat Rev. 2016;42:47-55. doi:10.1016/j.ctrv.2015.11.003
24. Zhao Y, Zhang YN, Wang KT, Chen L. Lenvatinib for hepatocellular carcinoma: From preclinical mechanisms to anti-cancer therapy. Biochim Biophys Acta Rev Cancer. 2020;1874(1):188391. doi:10.1016/j.bbcan.2020.188391
25. Fallahi P, Ferrari SM, Santini F, et al. Sorafenib and thyroid cancer. BioDrugs. 2013;27(6):615-628. doi:10.1007/s40259-013-0049-y
26. Grandinetti CA, Goldspiel BR. Sorafenib and sunitinib: novel targeted therapies for renal cell cancer. Pharmacotherapy. 2007;27(8):1125-1144. doi:10.1592/phco.27.8.1125
27. Gershenson DM, Miller A, Brady WE, et al. Trametinib versus standard of care in patients with recurrent low-grade serous ovarian cancer (GOG 281/LOGS): an international, randomised, open-label, multicentre, phase 2/3 trial. Lancet. 2022;399(10324):541-553. doi:10.1016/S0140-6736(21)02175-9
28. Planchard D, Smit EF, Groen HJM, et al. Dabrafenib plus trametinib in patients with previously untreated BRAFV600E-mutant metastatic non-small-cell lung cancer: an open-label, phase 2 trial. Lancet Oncol. 2017;18(10):1307-1316. doi:10.1016/S1470-2045(17)30679-4
29. de Bono J, Mateo J, Fizazi K, et al. Olaparib for Metastatic Castration-Resistant Prostate Cancer. N Engl J Med. 2020;382(22):2091-2102. doi:10.1056/NEJMoa1911440
30. Tutt ANJ, Garber JE, Kaufman B, et al. Adjuvant Olaparib for Patients with BRCA1- or BRCA2-Mutated Breast Cancer. N Engl J Med. 2021;384(25):2394-2405. doi:10.1056/NEJMoa2105215
31. Monk BJ, Parkinson C, Lim MC, et al. A Randomized, Phase III Trial to Evaluate Rucaparib Monotherapy as Maintenance Treatment in Patients With Newly Diagnosed Ovarian Cancer (ATHENA-MONO/GOG-3020/ENGOT-ov45). J Clin Oncol. 2022;40(34):3952-3964. doi:10.1200/JCO.22.01003
32. Fizazi K, Piulats JM, Reaume MN, et al. Rucaparib or Physician's Choice in Metastatic Prostate Cancer. N Engl J Med. 2023;388(8):719-732. doi:10.1056/NEJMoa2214676
33. Lee YC, Wong WT, Li LH, et al. Ginsenoside M1 Induces Apoptosis and Inhibits the Migration of Human Oral Cancer Cells. Int J Mol Sci. 2020;21(24):9704. Published 2020 Dec 19. doi:10.3390/ijms21249704
34. Tang YC, Zhang Y, Zhou J, et al. Ginsenoside Rg3 targets cancer stem cells and tumor angiogenesis to inhibit colorectal cancer progression in vivo. Int J Oncol. 2018;52(1):127-138. doi:10.3892/ijo.2017.4183

| **Primer** | **Forward（5'→3'）** | **Reverse（5'→3'）** |
| --- | --- | --- |
| CXCL12 | CTCAACACTCCAAACTGTGCCC | CTCCAGGTACTCCTGAATCCAC |
| CXCR4 | CTCCTCTTTGTCATCACGCTTCC | GGATGAGGACACTGCTGTAGAG |
| NES | TCAAGATGTCCCTCAGCCTGGA | AAGCTGAGGGAAGTCTTGGAGC |
| MYBL2 | CACCAGAAACGAGCCTGCCTTA | CTCAGGTCACACCAAGCATCAG |
| PBX3 | CCAGTGAAGAAGCCAAAGAGGAG | CAGCATAGAGGTTGGCTTCTTCC |
| REPS2 | CCTCCAACTCTGCAGCCAGAAT | CCATCCGATTCAAGTCACGAGG |
| GAPDH | GTCTCCTCTGACTTCAACAGCG | ACCACCCTGTTGCTGTAGCCAA |

**Table 5.** The qRT-PCR primer sequences used in this study
