# Supplementary material for: Biomimetic 3D-Bioprinted organoids of thymic epithelial tumors for translational drug screening and biomarker identification
Source: Mater Today Bio. 2026 Feb 11;37:102878. doi: 10.1016/j.mtbio.2026.102878 (PMC12924735; doi:10.1016/j.mtbio.2026.102878)
Supplement: Multimedia component 2 [file mmc2.docx]

**Supplementary Figures**


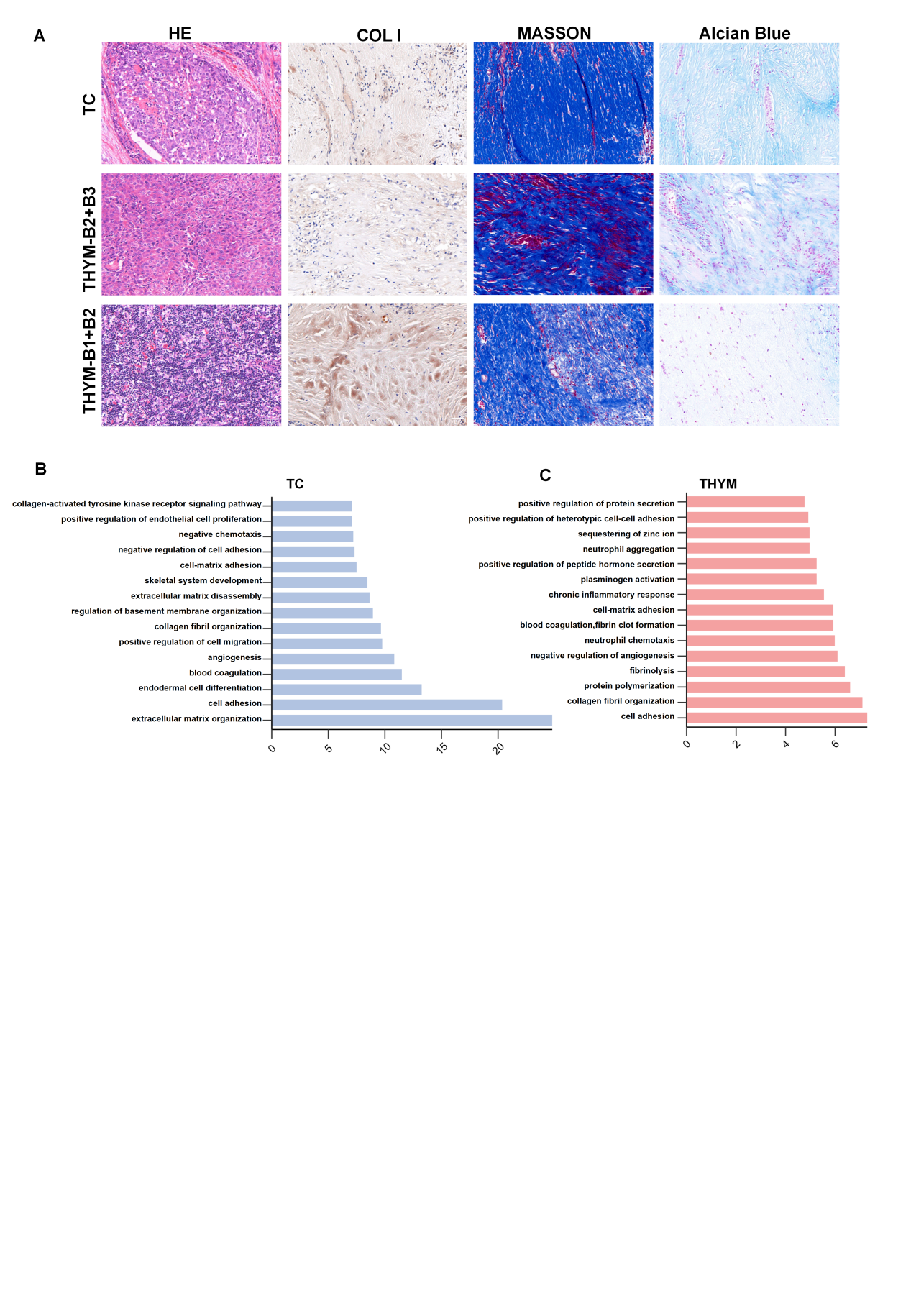


**Supplementary Figure 1.** (A) Histological and immunohistochemical staining of TC-squamous cell carcinoma and THYM-B type tissues, including hematoxylin and eosin (HE), collagen type I (COL I), Masson’s trichrome, and Alcian Blue staining.Scale bars: 50 μm. (B) Top 10 pathways in TC proteomics enrichment analysis.(C) Top 10 pathways in THYM proteomics enrichment analysis.


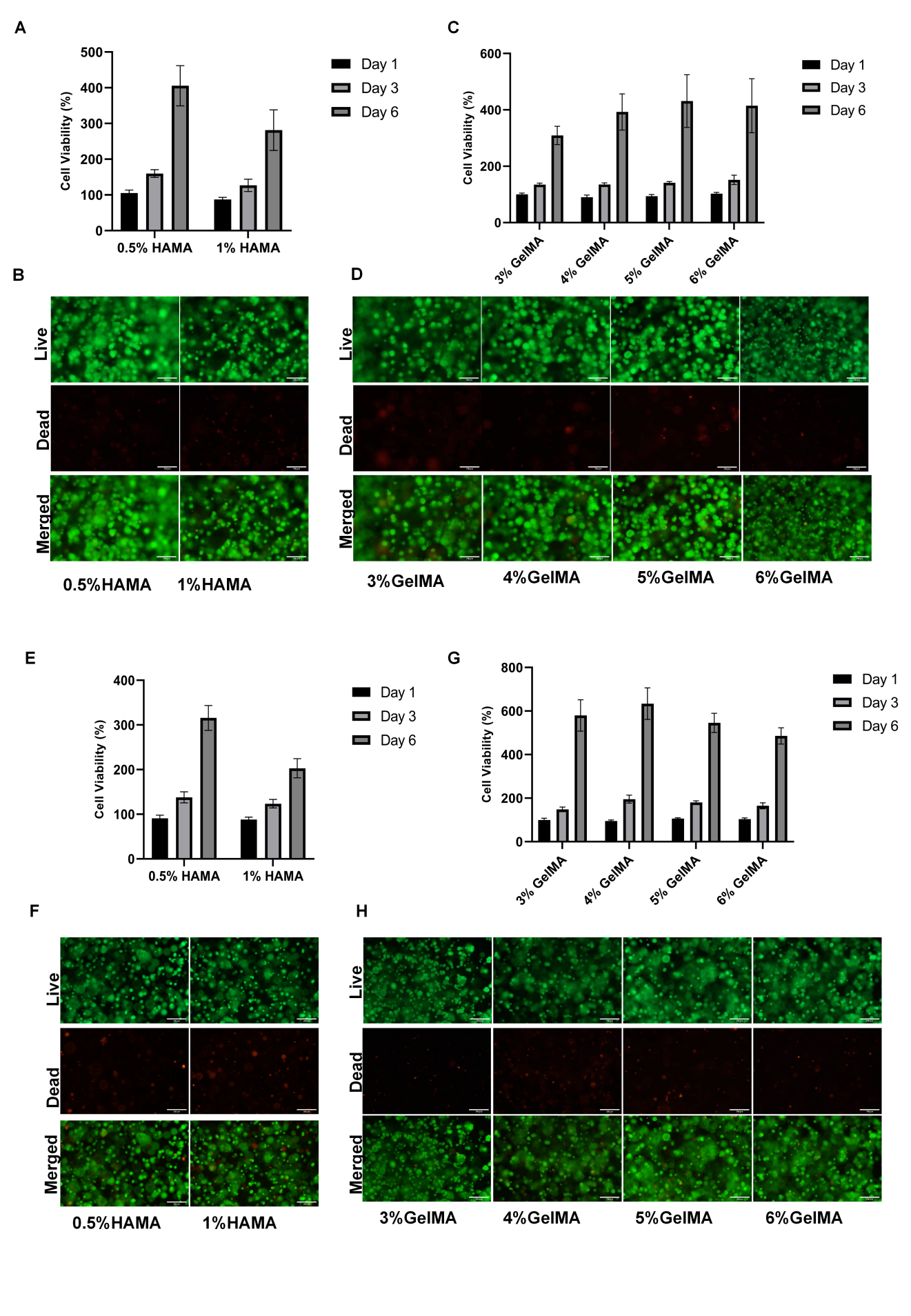


**Supplementary Figure 2.** Cell viability of IU-TAB-1 (A–D) and Ty-82 (E–H) cells in 3D-bioprinted constructs (HAMA: 0.5%, 1% [A,B,E,F]; GelMA: 3%,4%,5%,6% [C,D,G,H]) was quantified via CTG assay at Day 1, 3, 6, and visualized by live/dead fluorescence staining at Day 6.Scale bars:10x-200μm.


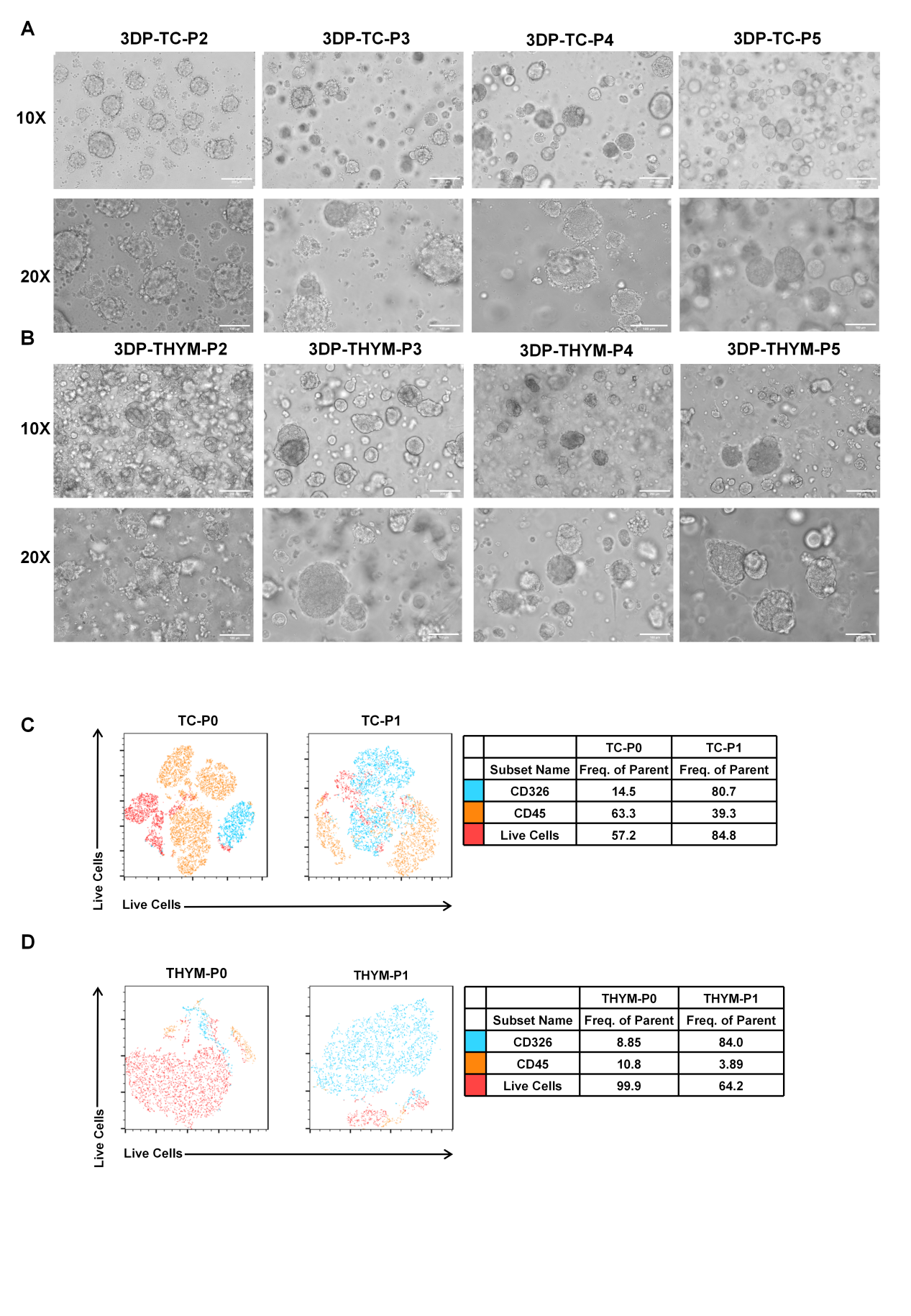


**Supplementary Figure 3.** (A-B) Representative bright-field images showing the morphology of 3DP TC and THYM organoids from passages 2 (P2) to 5 (P5) .Scale bars: 10x-200 μm ;20x-100μm.(C) t-SNE(Stochastic Neighbor Embedding) visualization of TC-P0 and TC-P1 flow cytometry data analysis. (D) t-SNE(Stochastic Neighbor Embedding) visualization of THYM-P0 and THYM-P1 flow cytometry data analysis.


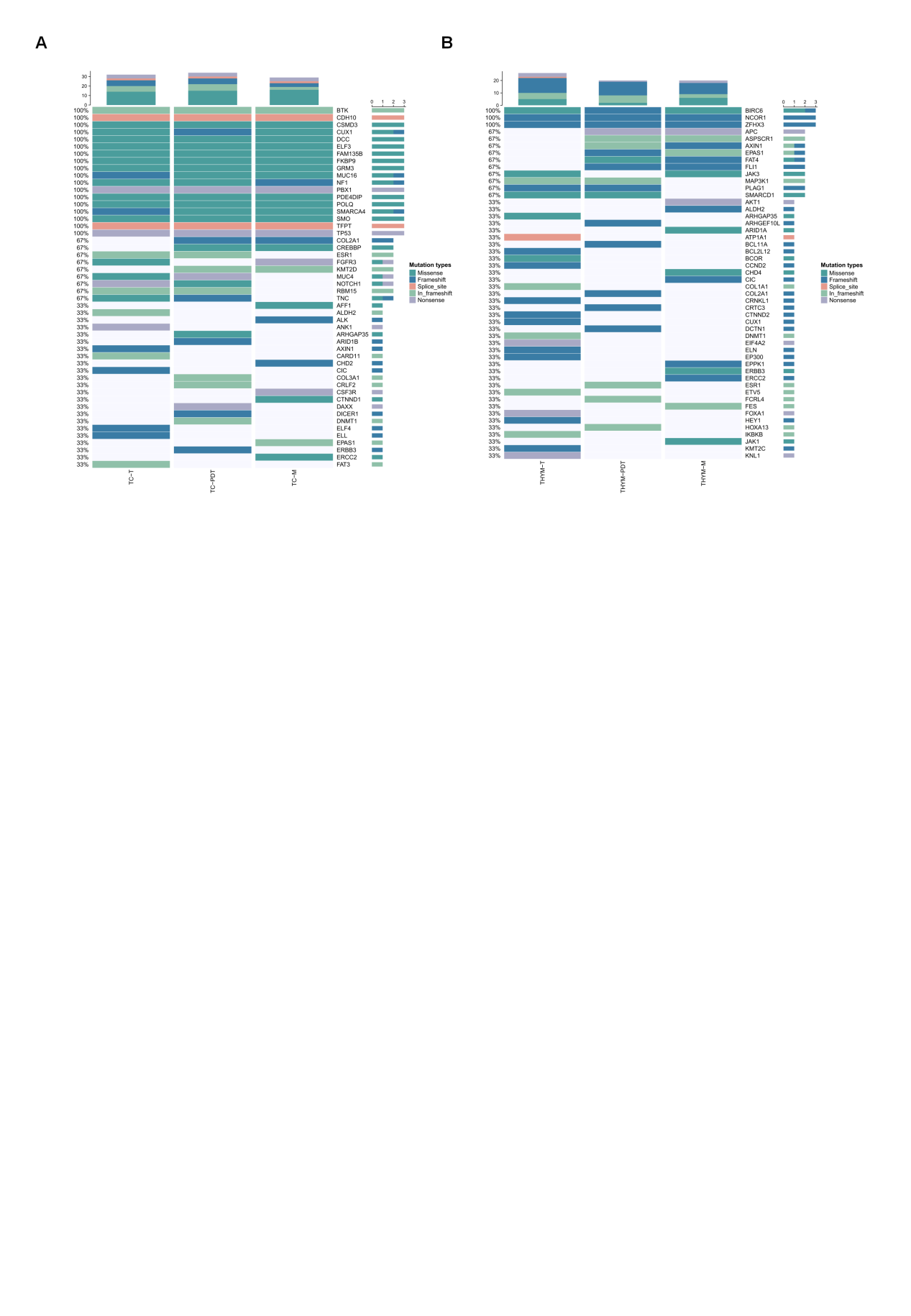


**Supplementary Figure 4.** (A) CNVs profiles of TC-Tumor,3DP-Oragnoid,Matrigel-Oragnoid.(B) CNVs profiles of THYM-Tumor,3DP-Oragnoid,Matrigel-Oragnoid


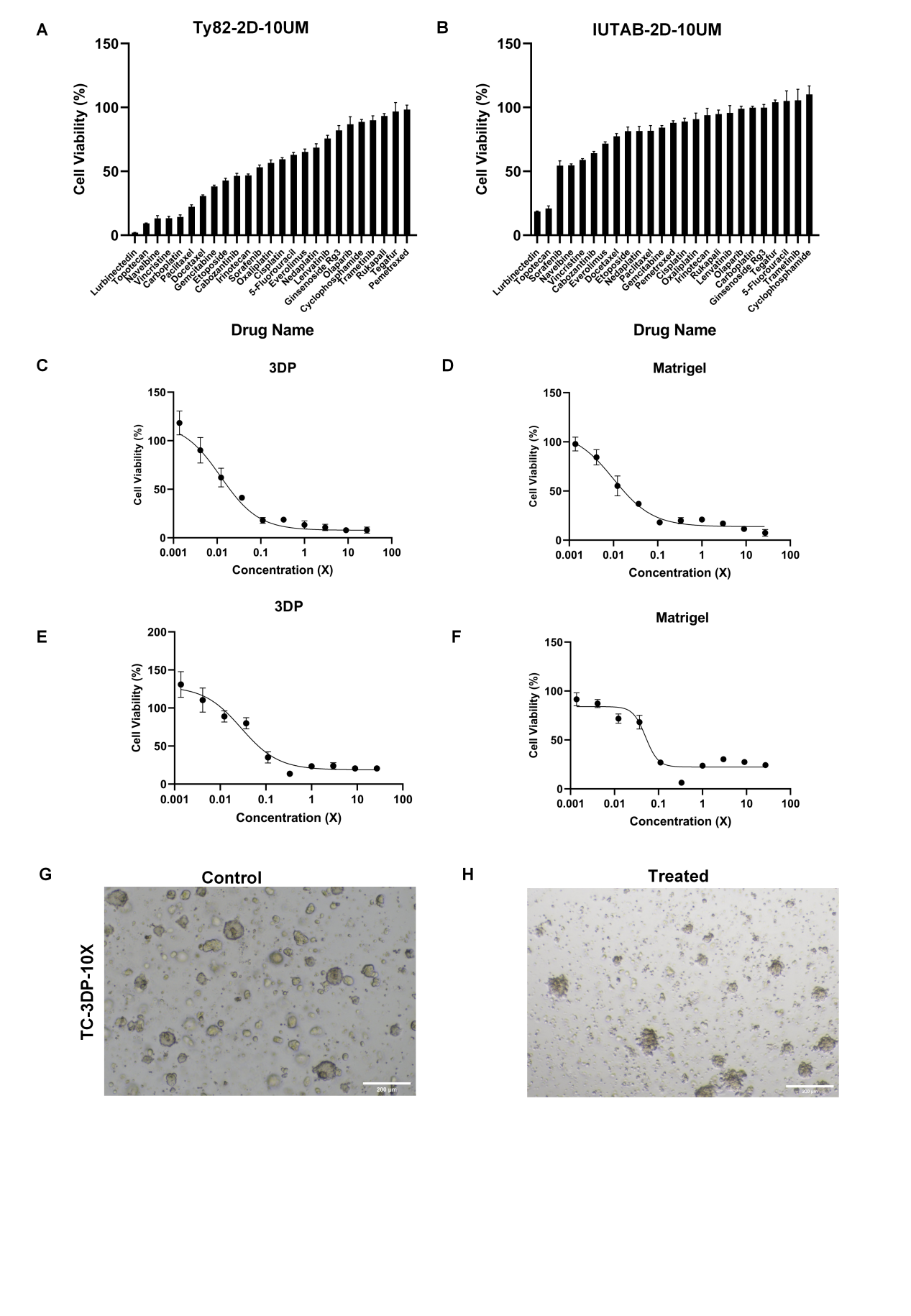


**Supplementary Figure 5.** (A-B) Comparative bright-field imaging of Ty-82 cell lines before and after drug treatment in 3D culture system. (C-F) Dose-response curves of TY82 cells treated with lurbinectedin, cultured in 3D bioprinted (3DP) constructs (C) and Matrigel (D), respectively. Dose-response curves of IU-TAB-1 cells treated with lurbinectedin, cultured in 3D bioprinted (3DP) constructs (E) and Matrigel (F), respectively. Cell viability was quantified across a gradient of lurbinectedin concentrations. (G-H) Comparative bright-field imaging of TC-Oragnoid before and after drug treatment in 3D culture system,Scale bars: 10x-200 μm.


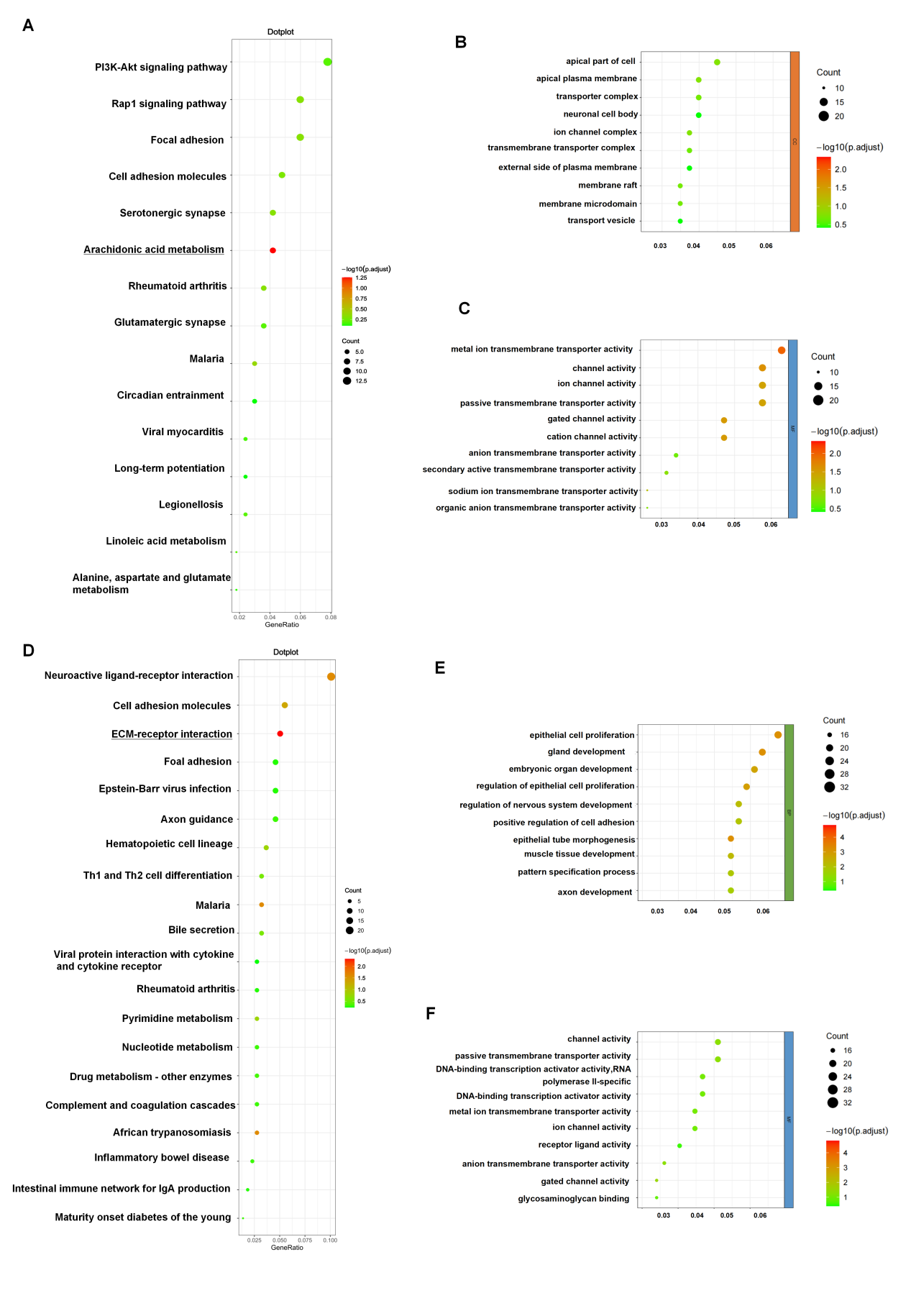


**Supplementary Figure 6.**(A) KEGG enrichment of downregulated pathways of Ty82. (B-C) Ty82 GO downregulated pathways enrichment of Cellular Component(CC) and Molecular Function(MF). (D) KEGG enrichment of upregulated pathways of Ty-82. (E-F) Ty82 GO upregulated pathways enrichment of Biological Process(BP) and Molecular Function(MF).


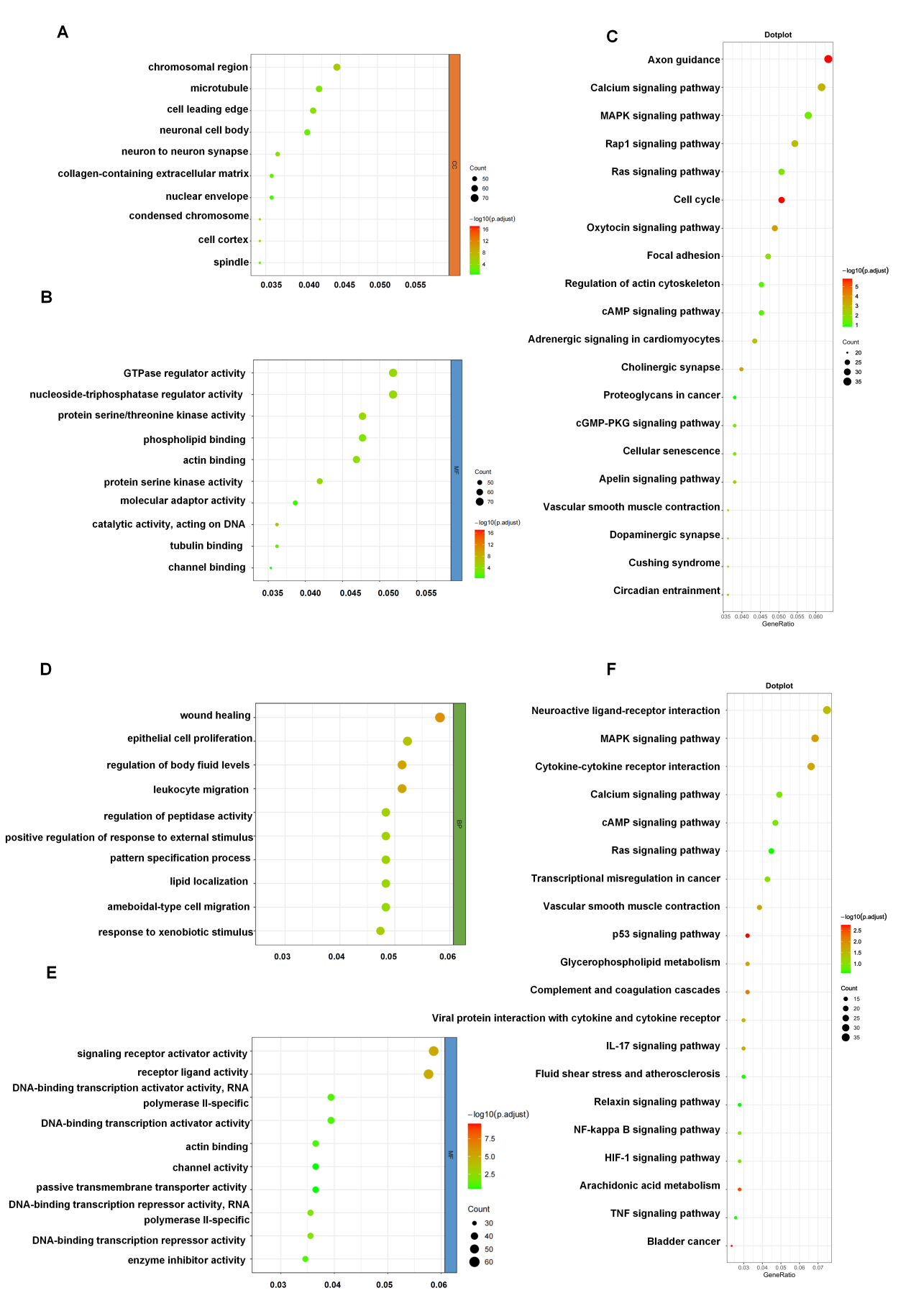


**Supplementary Figure 7.** (A-B) IU-TAB-1 GO downregulated pathways enrichment of Cellular Component(CC) and Molecular Function(MF). (C) KEGG enrichment of upregulated pathways of IU-TAB-1. (D-E) IU-TAB-1 GO upregulated pathways enrichment of Biological Process(BP) and Molecular Function(MF). (F) KEGG enrichment of upregulated pathways of IU-TAB-1.


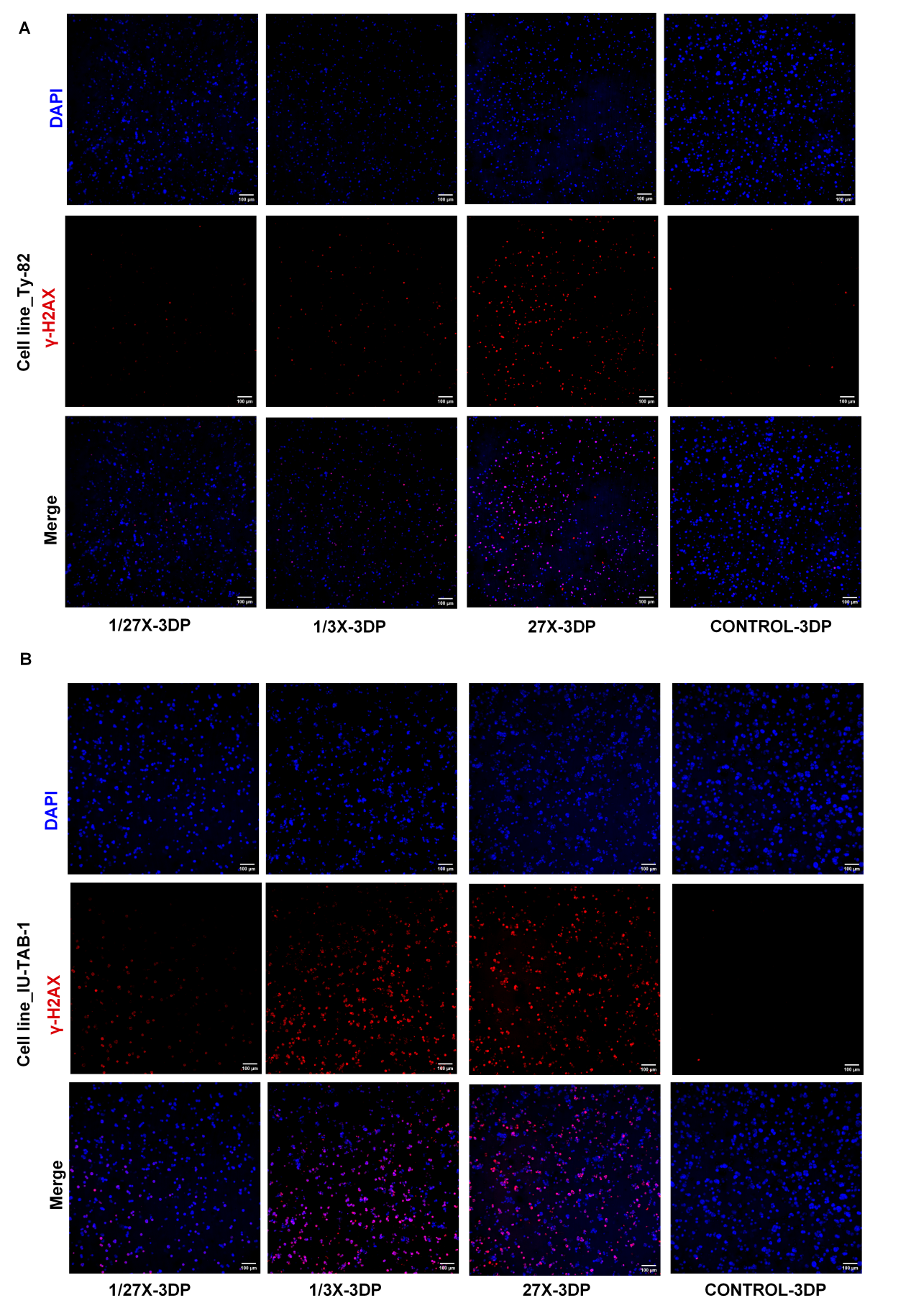


**Supplementary Figure 8A-B:** Immunofluorescence staining of γ-H2AX in IU-TAB cells cultured in 3D-bioprinted (3DP) constructs, treated with gradient concentrations of lurbinectedin 1/27X, 1/3X, 27X (X=Cmax of lurbinectedin)or control (untreated), Scale bars:20x-100μm.
